# Process optimization for biosynthesis of mono and bimetallic alloy nanoparticle catalysts for degradation of dyes in individual and ternary mixture

**DOI:** 10.1038/s41598-019-57097-0

**Published:** 2020-01-14

**Authors:** Sabyasachi Ghosh, Swarup Roy, Jishu Naskar, Ramen Kumar Kole

**Affiliations:** 1 Department of Biochemistry and Biophysics, University of Kalyani, Kalyani, Nadia, 741235 West Bengal India; 2 BioNanocomposite Research Center, Department of Food and Nutrition, Kyung Hee University, 26 Kyungheedae–ro, Dongdaemun–gu, Seoul, 02447 Republic of Korea; 3 Department of Agricultural Chemicals, Bidhan Chandra Krishi Viswavidyalaya, Mohanpur, Nadia, 741252 West Bengal India

**Keywords:** Biosynthesis, Catalyst synthesis, Nanoparticles

## Abstract

Nanoparticle (NP) catalysts are widely used for removal of dyes for single use, but there is an acute need for developing catalysts with high efficiency and reusability for mixed dyes. Here we first optimized the process (reactant proportion, temperature, time, and pH) for biosynthesis of monometallic Ag, Au and bimetallic Au–Ag alloy NP catalysts using *Polyalthia longifolia* leaf extract. The biosynthesized NP catalysts were characterized by UV-vis, DLS, Zeta potential, TEM and EDX study while the probable biomolecules responsible for biosynthesis were identified by FTIR and GC-MS/MS analysis. The NPs are found to be mostly spherical in shape (size 5–20 nm) with prolonged stability. We evaluated their chemo-catalytic performance through degradation of dyes (methyl orange, methyl violet, methylene blue) in individual and ternary mixture in presence of NaBH_4_. The degradation percentage (80.06–96.59% within 5 min), degradation kinetics (k = 0.361–1.518 min^–1^), half-life (T_50_ = 0.457–1.920 min) and 80% degradation (T_80_ = 1.060–4.458 min) of dyes indicated highest catalytic activity of alloy in ternary mixture. Here we report a unique vacuum filtration system using alloy coated beads with excellent catalytic activity which could be reused thrice for removal of hazardous ternary mixed dyes with great promise for environmental remediation.

## Introduction

Over a decade, catalysis plays a prominent role for more than 90% of the chemicals manufacturing process in institutional research and industrial applications such as chemical, energy, pharmaceutical, polymers and in the protection of our environment^1–3^. The exciting improvement on nanoparticle (NP) catalysts and their potential applications for the removal of dyes from different types of wastewater has gained remarkable scientific attention in the field of catalysis research^3–5^. Industrial wastewater would be possible to contain more than one kind of various organic dyes, among them methyl orange (MO), methyl violet (MV) and methylene blue (MB) can contribute a lot to worldwide environmental pollution^6,7^. Metallic NPs based catalytic process has shown a great potential for the degradation or removal of dyes due to their smaller size, large surface-to-volume ratios, huge number of active sites, strong electron transfer abilities, high efficiency and take less time to degrade dye^4,5^. Among different noble metals NPs, especially AuNPs and AgNPs are well known ones used as effective catalysts in inorganic and organic reactions^5,8^. Particularly in the catalytic reaction process, bimetallic NPs often exhibit higher catalytic activity and selectivity than their monometallic counterparts^9^. Though, the recovery and reusability of the homogeneous catalysts is extremely difficult and often requires tedious process^1,4,10^. Apparently, there is an acute need for developing a new type of catalyst that process high efficiency and reusability.

Numerous chemical, physical and biological methods are available for synthesis of noble metal NPs but these methods have some inherent advantages and disadvantages. In general, some of the reducing, stabilizing agents and solvents used in the chemical methods are found to be toxic and in physical synthesis methods large amounts of energy are required to maintain the high pressure and temperature conditions for synthesis^11–13^. Of late, biosynthesis methods have some shortcoming such as adherence of organism on the surface of NPs make chances of infection, tedious process of isolation technique and maintenance of microbial culture in the case of using microorganisms (such as algae, bacteria, fungi, etc.)^14–17^. Biosynthesis of NPs using plants is certainly a better option than other biological method because of less or no chances of contamination, cost-effective and requirement of very simple laboratory setup for NPs production. Also, plant mediated biosynthesis are simple, safe to handle, single-step, rapid, improved stability and suitable for large-scale production^18,19^.

*Polyalthia longifolia* is a lofty, straight columnar and evergreen plant with horizontal short branches. It is a multipurpose tree whose extracts and isolated compounds have been studied for various biological activities^20–22^. To explore further the aqueous leaf extract of *P. longifolia* was utilized for biosynthesis of NPs of novel monometallic (Au, Ag) and bimetallic (Au–Ag) alloy. Various physico–chemical parameters of NPs were systematically studied by chromatographic, spectroscopic and microscopic method. Their chemo-catalytic activity and reusability of NPs for degradation of dyes in aqueous solution has also been investigated.

## Results and Discussion

### Synthesis and process optimization

**Figure 1:**
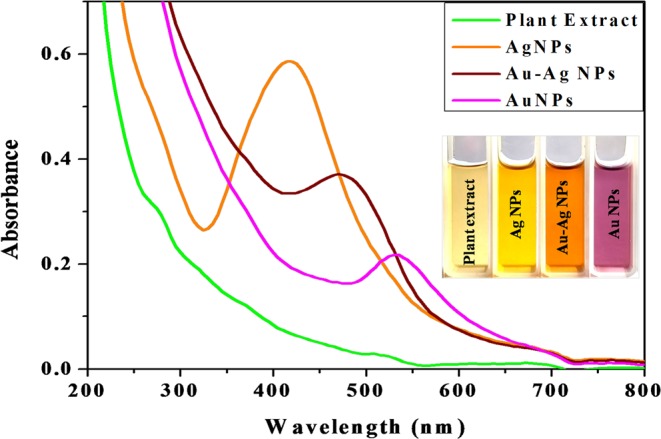
UV–vis absorbance spectra of the plant extract, synthesized Ag, Au and Au–Ag (1:1) alloy NPs. Ag, Au and Au–Ag (1:1) alloy NPs shows distinct absorbance peak at (λ_max_) 415 nm, 535 nm and 470 nm, respectively. Inset shows the visual color of the nanoparticles solution after biosynthesis using plant extract solution.

The formation of mono-metallic Ag, Au and bimetallic Au–Ag NP catalysts under the present experimentation could be visualised by initial color change of the reaction mixtures from pale yellow to golden yellow, dark-purple and reddish brown, respectively (Fig. 1) and the reaction was completed within 1 h. AuNPs and AgNPs exhibit surface plasmon resonance (SPR) band due to collective oscillation of the conduction free band electrons of the metal in presence of incident photon^19^. SPR band intensity depends on the type of NPs, morphology, composition, and surrounding medium^23^. Synthesis of metal NPs were detected by such type of specific SPR bands. Therefore, the formation of Ag, Au and Au–Ag NPs was further confirmed by UV–vis spectrophotometer which shows distinct absorbance peak (λ_max_) at 415 nm, 535 nm and 470 nm, respectively (Fig. 1) due to NPs SPR. Therefore, the mono-metallic Ag, Au and bimetallic Au–Ag NPs were successfully synthesized using *P. longifolia* leaf extract to act as both reducing and capping agent; thus, no extra reductant or surfactant agent added. Moreover, UV–vis spectroscopy is also one of the major characterization methods to investigate the nature of synthesized bimetallic Au–Ag NPs. Core–shell NPs usually show two SPR absorption peaks and alloy type NPs show single SPR peak usually appears in between the SPR peaks of two individual pure metals^24,25^. The bimetallic Au–Ag NPs appeared single SPR peak in between 535 and 415 nm (i.e. SPR peaks position of AuNPs and AgNPs) indicating the formation of alloy type bimetallic Au–Ag NPs. Alloy type bimetallic NPs are produced due to the identical lattice constants of Au and Ag that facilitate their homogeneous distribution within the volume of the particle^19,24^.

**Figure 2:**
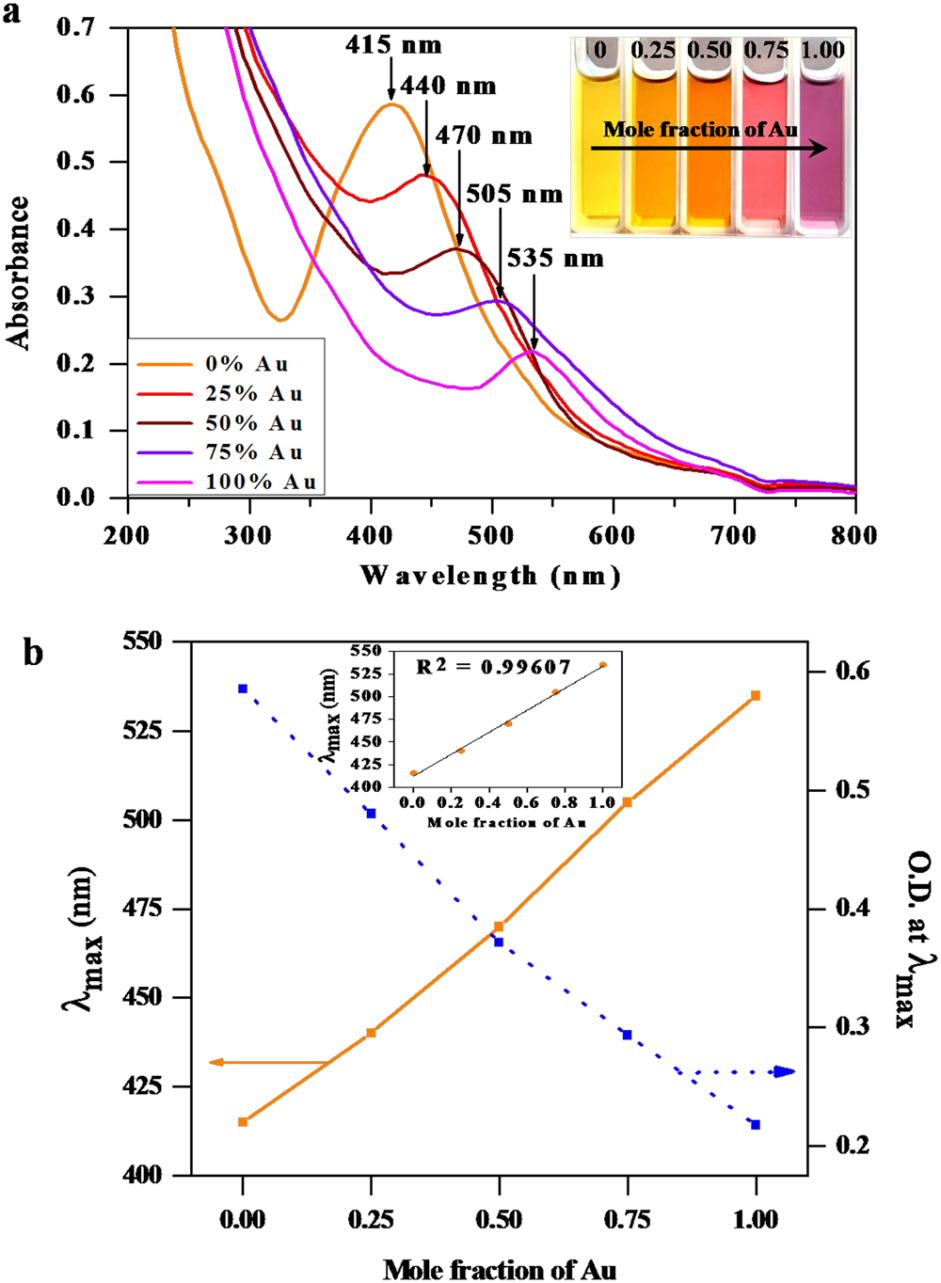
(**a**) Effect of molar ratio (Au:Ag) on SPR of biosynthesized Au-Ag bimetallic NPs. Inset shows the corresponding visual color change of synthesized bimetallic NPs at various molar ratio of Au:Ag. (**b**) Variation of λ_max_ and O.D. at λ_max_ of the biosynthesized Au–Ag bimetallic NPs with mole fraction of Au. Inset shows the linear regression plot of the λ_max_ vs. mole fraction of Au.

Bimetallic Au-Ag alloy NPs were synthesized at pH 12.0 to investigate the effect of the ratio of the precursor salts on nature of synthesized bimetallic alloy NPs. The SPR peaks of different Au–Ag compositions (1:0), (0.75:0.25), (0.50:0.50), (0.25:0.75) and (0:1) were observed at ~535, 505, 470, 440 and 415 nm, respectively (Fig. 2a). Figure 2a (inset) shows the color changes of different Au–Ag NPs products. The quasi linear dependence of SPR peak with Au molar ratio indicating that the SPR peak linearly increases (R^2^ = 0.99607) as the Au molar ratio increased (Inset Fig. 2b). Figure 2b displays the relationship between plot of λ_max_ vs. mole fraction of Au and O.D. at λ_max_ vs. mole fraction of Au. The SPR peak (λ_max_) of Au–Ag NPs shifted from 415 to 535 nm with increasing Au mole fraction. However, the SPR peak intensity decreases in Au from mole fraction 0 to 1.0 signifying that the strong alkaline medium is not suitable for the reduction of Au^3+^.

**Figure 3:**
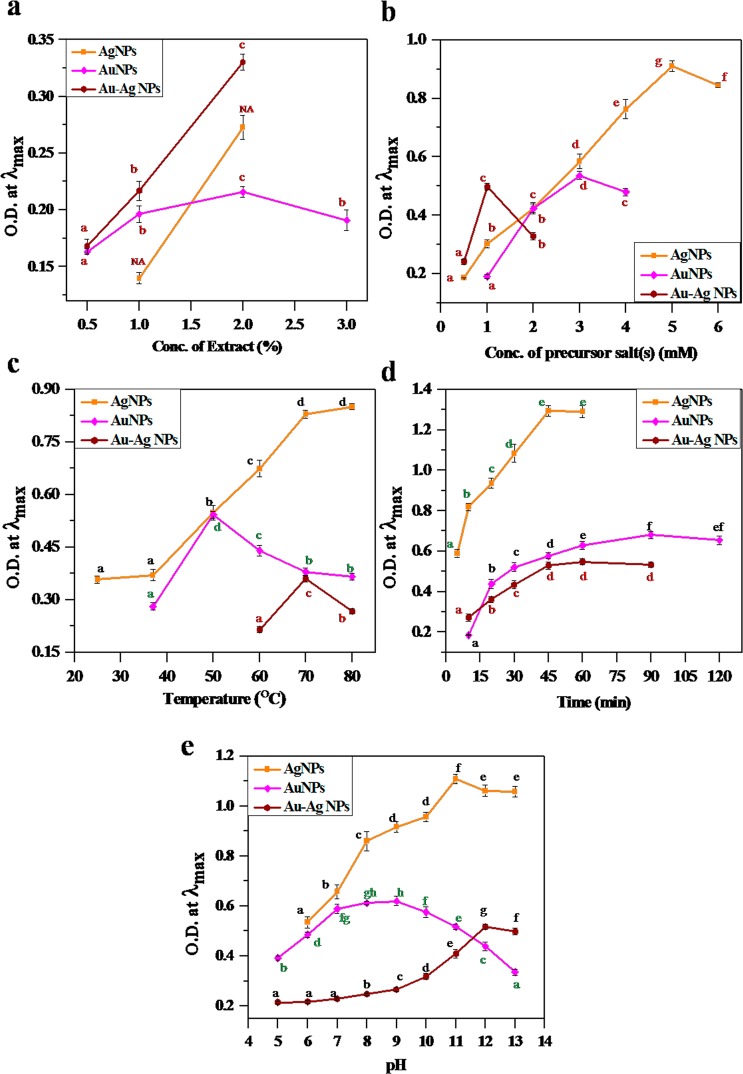
Effect of (**a)** Plant extract concentration, (**b**) Precursor salt(s) concentration, (**c**) Temperature, (**d**) Time and (**e**) pH on O.D. at λ_max_ of the synthesized Ag, Au and Au-Ag (1:1) NPs. λ_max_ for Ag, Au and Au-Ag (1:1) NPs at 415 nm, 535 nm and 470 nm, respectively. Data were represented as mean ± standard deviation and any two means on the same colored point with the same letter are not significantly different (*p* > 0.05). NA = Not Applicable i.e. Not Determined.

The biosynthesis method of NPs was optimized by controlling various parameters. The effect of each parameter (viz. concentration of extract, concentration of salt(s), temperature, reaction time and pH) for the formation of metallic NPs was assessed using UV-vis spectroscopy (Figs. S1–5) and suitable conditions were proposed (see detail in Supplementary Note) to obtain highest intensity at λ_max_. A wide range of color change was observed for formation of the NPs at various conditions (Figs. S1–5). The optimum physico-Chemical conditions for synthesis of the NPs were varied according to the type of metal NPs as displayed in Fig. 3(a–e). The process for synthesis of all NPs has been optimised with plant extract concentration of 2% in aqueous media under dark condition (Fig. 3a). The other physico-chemical conditions for AuNPs were optimized using 3 mM salt (HAuCl_4_.H_2_O) solution at 50 °C for 75 min under pH 8.0 (Fig. 3b–e). For AgNPs, 5 mM of AgNO_3_ and heating at 80 °C for 45 min under pH 12.0 (Fig. 3b–e) and the synthesis of bimetallic alloy NPs were optimised as 1 mM (0.5 mM each of HAuCl_4_.H_2_O and AgNO_3_) solution at 70 °C for 60 min under pH 12.0 (Figs. 2 and 3b–e).

### Characterization of nanoparticle catalysts

#### DLS analysis and zeta potential measurement

The average particle size (Z_avg._) and size distribution profile of the NP catalysts were measured using Dynamic light scattering (DLS). The Z_avg._ values were 44.9, 47.47 and 70.98 nm for the biosynthesized Ag, Au and Au–Ag alloy NPs, respectively. The size distribution profile of stable NPs in suspension is presented in Fig. S6. The polydispersity index (PDI) was 0.505, 0.299 and 0.392 for Ag, Au and Au–Ag alloy NPs, respectively. The PDI value ≤ 0.1 is considered to highly monodisperse while values of 0.1–0.4 and more than 0.4 are considered to moderately and highly polydisperse, respectively^26^. A comparison of the PDI value and average particle size of the NPs indicated that this process produced smallest NPs with polydispersity but to confirm the shape and size of the prepared nanoparticles we have studied TEM in the following section.

The zeta potentials (ZP) of the biosynthesized NPs (Ag: −21.3, Au: −15.5 and Au–Ag alloy: −22.5 mV) are shown in Fig. S7. The NPs with ZP from −10 and +10 mV are considered to neutral. NPs having ZP more positive than +30 mV or more negative than −30 mV are generally considered as stable^27^. However, under the present experimentation the biosynthesized NPs (with ZP > −15 mV) were found to be quite stable for three weeks as indicated by UV−vis spectra signifying stable size (no aggregation). The repulsive energy due to high negative charge on the surface prevents aggregation of the NPs^28,29^.

#### TEM and EDX analysis

**Figure 4:**
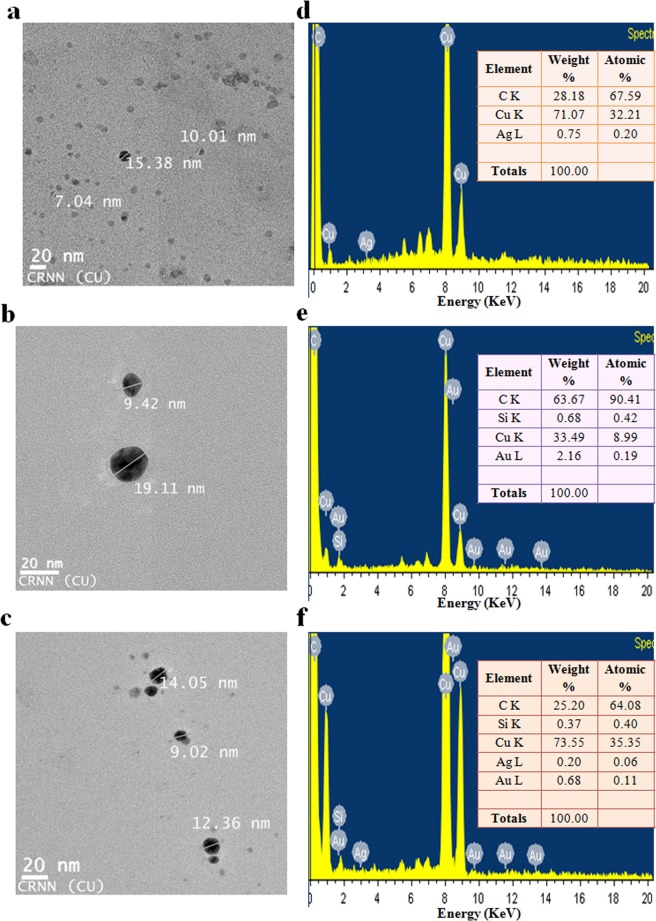
TEM images of the biosynthesized (**a**) Ag, (**b**) Au and (**c**) Au–Ag alloy NPs. EDX spectra of the biosynthesized (**d**) Ag, (**e**) Au and (**f**) Au–Ag alloy NPs; the inset shows the elemental analysis of the corresponding Ag, Au and Au–Ag alloy NPs.

Transform electron microscopy (TEM) images of the NPs (Fig. 4a–c) reveals particle size within the range of 5–20 nm for AgNPs, 5–20 nm for AuNPs and 5–15 nm for Au–Ag NPs. The particles were most by spherical in shape with uniform size distribution. It is noteworthy that synthesized NPs are well separated from each other signifying the absence of aggregation. In addition to repulsion of NPs due to similar charges, the presence of phytochemicals surrounding the metal NPs might also be preventing aggregation of NPs^19,30^. TEM image (Fig. 4c) of the synthesized bimetallic NPs further indicated that there is a homogeneous contrast for each kind of NP, thereby, suggesting that the electron density was uniformly distributed within the volume of the NP. So, the bimetallic NPs appeared as bimetallic alloy NPs. The results are in strong agreement with those observed using UV–vis absorbance data (Fig. 1). Similar observation has also been reportedearlier^19,31^. Energy dispersive X–ray Spectroscopy (EDX) spectrum of biosynthesized AgNPs (Fig. 4d) and AuNPs (Fig. 4e) illustrated the presence of signals of Ag and Au element, respectively. Although there are background signals is appeared for Cu, C and Si impurity which may be due to use of copper grid, carbon coating on the copper grid, whereas the rest of the elements may be due to biomolecules (carbon including) from the plant extract that attached to NPs. The EDX spectrum of Au–Ag NPs (Fig. 4f) indicates the presence of Au and Ag elements.

### Detection of functional groups and biomolecules

#### FTIR analysis

Fourier transform electron spectroscopy (FTIR) spectra of aqueous leaf extract and metal NPs are shown in Fig. S8 and the essential FTIR absorption frequencies representative data are shown in Table S1. The broad FTIR bands appeared in the region from 3409 to 3568 cm^−1^ corresponding to O–H stretching vibration of phenols^32,33^. The strong absorption bands around 1617–1620 cm^−1^ correspond to the bending vibration of N–H groups^34^. The absorption peaks positioned at 1399 cm^−1^ and 1535 cm^−1^ are attributed to the bending vibration of N=O groups and stretching vibration modes of C=O functional groups associated with amide groups, respectively^33,35^. The band range from 1115–1279 cm^−1^ corresponds to stretching vibration of C=O in ester and carboxylic acids, respectively^34–36^. In addition, very strong absorption band around 1035–1068 cm^–1^ indicates the presence of stretching frequency of C=O functional groups linked with carboxylic acids, ester and alcohols of biomolecules^34^. Interestingly, most of the functional groups present in plant biomolecules have also been detected in the bio-synthesized NPs. Therefore, the biomolecules might have acted the dual functions for synthesis and stabilization of the NPs in aqueous solution. We conclude from the overall observations that the reduction and stabilization of Ag, Au and Au–Ag NPs could be due to the presence of some functional groups such as –OH, –NH, –CHO, –COOH and –COOR^35,37^.

#### GC-MS/MS analysis

The plant biomolecules were identified in extract (before and after formation of NPs) through tandem mass spectrometry (MS/MS) attached with Gas Chromatography (GC). The mass spectra of these compounds were clearly matched with those found in the NIST spectral library. Although analytical standards were not used for confirmative identification of compounds, but the represented results may be considered rational with the probability of correct identification of major compounds (Table S2). For example, many of the components identified, including azulene (retention time, RT: 11.47 min) and eicosane (RT: 35.07 min) have been previously reported as important components in *Polyalthia*^38,39^. Several alcohols were also found with higher probability in the extract like Benzyl alcohol (RT: 9.46 min), Phenylethyl Alcohol (RT: 10.19 min), and 2-Methoxy-4-vinylphenol (RT: 13.55 min). A number of esters were also found in the samples including hexanedioic acid, dimethyl ester (RT: 12.96 min), pentanedioic acid, dimethyl ester (RT: 15.05 min), and decanoic acid, decyl ester (RT: 25.42 min). Chromatograms for all samples are provided as Fig. S9. Therefore, the FTIR detected functional groups of these compounds might be contributed for the reduction and stabilization of biosynthesized NPs.

### Catalytic activity of nanoparticles

**Figure 5:**
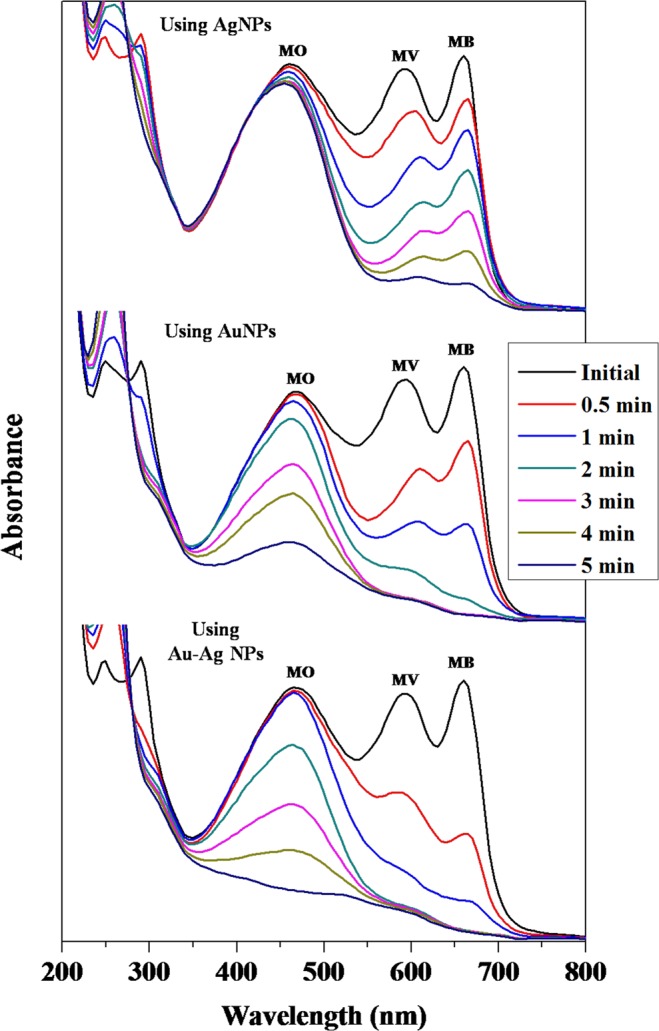
Time-dependent UV–vis spectra for degradation of mixed (MO+ MV+ MB) dye by NaBH_4_ in presence of various nanoparticle as catalyst. Catalysts used are shown in the uper-left side of each degradation profile.

The catalytic action of the metal NPs on degradation of methyl orange (MO), methyl violet (MV) and methylene blue (MB) individually and also in ternary mixture was evaluated by measuring the residual concentration of dyes spectrophotometrically at 470, 585 and 665 nm, respectivelyat different time intervals (Fig. S10a–c). There was no significant shifting of absorption peaks in ternary mixed dye solution (Fig. S10d). Therefore identification of individual dyes could be possible in ternary mixture. The catalytic degradation of various organic dyes by NaBH_4_ in presence of NPs is widely applied to evaluate the catalytic activity of various NPs. The degradation of dyes in presence of NaBH_4_ without addition of metals (for a long time) indicated a little or no change in the intensity of absorption due to dyes (Fig. S10). Therefore, the results indicated that the degradation of dyes by NaBH_4_ is thermodynamically favorable, but kinetically difficult and takes longer time or may not proceed in absence of any catalyst^40^. The degradation of dyes in solution was measured at constant interval of time in presence of NP as catalyst (Figs. 5 and S11a–c) and evaluated in terms of percent degradation and reaction rate.

#### Degradation percentage

The degradation pattern of the dyes in individual solution is shown in Fig. S12a–c and for the ternary mixture in Fig. 5. The percent degradation (using Eq. 1) of the dyes is presented in Table 1. The results demonstrate that Au-Ag alloy NPs with highest catalytic activity (96.59%) for degradation of MB after 5 min (in ternary mixture) in presence of NaBH_4_ (Table 1 and Fig. S12a). The catalytic degradation of MV and MO was also similar to that observed for MB. The degradation percent was higher in MV (86.88%, Fig. S12b) and for MO it was 80.06% (Fig. S12c) after 5 min in presence of Au-Ag alloy NPs in ternary mixture solution. The study indicated that the dye degradation performance of Au-Ag alloy NPs as a catalyst was much better compared to AuNPs and AgNPs (Table 1 and Fig. S12).

**Table 1:** Comparison of performance of biosynthesized nanoparticle catalysts towards dye degradation.

Nanoparticle catalysts	Parameters	Dye sample					
		Individual			In ternary mixture		
		MB	MV	MO	MB	MV	MO
AgNPs	% Degradation	52.93 ± 0.28	78.68 ± 0.52	15.38 ± 0.55	88.04 ± 0.24	86.54 ± 0.34	9.28 ± 0.39
	Time (min)	5	5	5	5	5	5
	k (min^−1^)	0.126 ± 0.002	0.281 ± 0.006	0.029 ± 0.001	0.422 ± 0.002	0.364 ± 0.004	0.015 ± 0.001
	T_50_ (min)	5.501 ± 0.076	2.467 ± 0.054	23.916 ± 0.826	1.642 ± 0.008	1.902 ± 0.018	45.238 ± 1.667
	T_80_ (min)	12.771 ± 0.177	5.728 ± 0.126	55.527 ± 1.916	3.813 ± 0.018	4.417 ± 0.043	107.267 ± 3.871
AuNPs	% Degradation	96.39 ± 0.21	59.70 ± 0.47	71.39 ± 0.24	96.13 ± 0.23	86.87 ± 0.32	59.35 ± 0.87
	Time (min)	5	5	5	5	5	5
	k (min^−1^)	0.641 ± 0.003	0.143 ± 0.003	0.242 ± 0.003	0.639 ± 0.005	0.310 ± 0.002	0.196 ± 0.004
	T_50_ (min)	1.081 ± 0.005	4.836 ± 0.085	2.868 ± 0.036	1.084 ± 0.008	2.238 ± 0.015	3.543 ± 0.082
	T_80_ (min)	2.510 ± 0.012	11.227 ± 0.196	6.659 ± 0.084	2.518 ± 0.018	5.196 ± 0.035	8.226 ± 0.190
Au–Ag NPs	% Degradation	94.4 8 ± 0.36	68.90 ± 0.32	77.57 ± 0.51	96.59 ± 0.11	86.88 ± 0.13	80.06 ± 0.67
	Time (min)	5	5	5	5	5	5
	k (min^−1^)	1^st^ 0.513 ± 0.016 2^nd^ 0.037 ± 0.003	0.183 ± 0.002	0.269 ± 0.003	1^st^ 1.518 ± 0.012 2^nd^ 0.095 ± 0.002	1^st^ 0.865 ± 0.008 2^nd^ 0.073 ± 0.006	0.361 ± 0.006
	T_50_ (min)	1^st^ 1.352 ± 0.042 2^nd^ 18.65 ± 1.538	3.787 ± 0.036	2.576 ± 0.028	1^st^ 0.457 ± 0.003 2^nd^ 7.323 ± 0.160	1^st^ 0.801 ± 0.007 2^nd^ 9.536 ± 0.786	1.920 ± 0.030
	T_80_ (min)	1^st^ 3.138 ± 0.097 2^nd^ 43.303 ± 3.573	8.793 ± 0.083	5.982 ± 0.067	1^st^ 1.060 ± 0.008 2^nd^ 17.002 ± 0.37	1^st^ 1.860 ± 0.018 2^nd^ 22.141 ± 1.826	4.458 ± 0.069

#### Degradation kinetics

The catalytic activity of the NPs was further evaluated in terms of reaction kinetics of the dye degradation (using Eq. 2) as shown in Fig. S13. The experimental data can be fitted in two stages for degradation of MB and MV (only in ternary mixture) by Au–Ag alloy NPs (Fig. S13a,b) and almost complete degradation happened in first stage upto 2 min. The straight line indicated the degradation reaction to follow pseudo–first–order kinetics^41^ with good fits linear regression coefficient (R^2^). The calculated kinetic rate constants (k) of dye degradation are shown in Table 1. The highest rate of degradation of MB was obtained in mixture solution (k = 1.518 min^−1^) in presence of Au–Ag alloy NPs compared to AuNPs (k = 0.639 min^−1^) and AgNPs (k = 0.422 min^−1^). The maximum degradation rate of MV (k = 0.865 min^−1^) and MO (k = 0.361 min^−1^) was observed in presence of Au-Ag alloy NPs as catalyst in mixture (Table 1). Among the NP catalysts Au-Ag alloy NPs appeared to be the most efficient for degradation of the dyes.

#### Half-life and time required for 80% degradation

The rate of dye degradation in catalytic reaction was also determined by the half-life (T_50_) and time required for 80% degradation (T_80_) using Eqs. 3 and 4, respectively as shown in Table 1. The calculated half-life for MB was found to be 0.457 min (catalysed by Au-Ag NPs), 1.084 min (catalysed by AuNPs) and 1.642 min (catalysed by AgNPs) in ternary mixture and 1.352, 1.081 and 5.501 min in single solution in presence of the respective NPs (Table 1). The half-life value for MV degradation (0.801–2.238 min in mixture and 2.467–4.836 min in individual solution) was comparable to MB. The corresponding values of MO were much higher (1.920–45.238 min) in mixture and (2.576–23.916 min) in individual solution (Table 1). Therefore, Au-Ag alloy NPs exhibited the highest catalytic activity compared to AuNPs and AgNP. The calculated T_80_ values of dyes catalysed by Au-Ag alloy NPs were found to be 1.060 min in case of MB, 1.860 min in MV and 4.458 min in MO in ternary mixture and 3.138, 8.793 and 5.982 min in individual solution, respectively (Table 1).

The organic dye degradation by sodium borohydride (NaBH_4_) in presence of metallic nanoparticle as catalyst has been widely investigated. BH_4_^−^ gets dissociate to produce H_2_ and electron in aqueous solutions^41^. The possible mechanism of dye reduction is electron relay effect produced by NPs and it involves NP induced transfer of electrons from BH_4_^−^ ion to organic dye compounds^41–43^. Though in all the experiments, equivalent quantity of dye and metal NPs was used, but the rate of degradation varied for different dyes. The difference in the rate of degradation may be depending upon NPs with smaller size, large volume to surface area ratio, maximum number of active sites and difference of work function values (eV) for three metals NPs and the chemical structure of the target dye^8,44–46^. Metallic NPs may also favor the reaction by reducing the activation energy, and thereby reducing kinetic barrier between the donor and the acceptor^8,46^. In addition, plant extract based synthesis of NPs could have additional catalytic activity as the surface of NPs is covered by plant biomolecules which may transfer matrix for chemical reaction and helps to increase the catalyzed reaction rates^13^. A comparative analysis of dye degradation under the present investigations with those reported earlier^6,44,47–53^ has been presented in Table 2. The comparison data revealed that in our work the degradation time was fastest in all case compared to the previously published data whereas catalytic reaction rate is either similar or improved in our present study. The results show that Au–Ag alloy NPs to possess promising catalytic activity in dyes (MB, MV and MO) degradation in ternary mixture.

**Table 2:** Comparative studies of several catalysts for the degradation of dyes as reported in the literature.

Dye		Catalysts	Method	Reagent/ source	% of Degradation	Degradation Time (min)	Rate Constant, k (min^−1^)	References
MB	Individual	AuNPs	Reduction	NaBH_4_	~100%	12	0.043	^6^
		Ag NPs	Photodecolorization	Sun Light	97%	150	0.021	^44^
		Au-Ag NPs	Photodecolorization	Sun Light	96%	90	0.040	^44^
		Au-Ag NPs	Reduction	NaBH_4_	94.48%	5	0.513	Present work
	In ternary mixture	Au-Ag NPs	Reduction	NaBH_4_	96.59%	5	1.518	Present work
MV		Ag Nps	Photodecolorization	Sun Light	85.56%	20	0.034	^47^
		Fe^0^@Guar gum-*crosslinked*-soya lecithin nanocomposite hydrogel	Photodecolorization	Sun Light and H_2_O_2_	81.00%	120	—	^48^
		ZnO NPs	Photodegradation	UV Lamp	87.00%	80	—	^49^
		Au-Ag NPs	Reduction	NaBH_4_	68.90%	5	0.183	Present work
	In ternary mixture	Au-Ag NPs	Reduction	NaBH_4_	86.88%	5	0.865	Present work
MO		AgNP-biophytum	Reduction	NaBH_4_	~100%	9	0.276	^50^
		AuNPs	Reduction	NaBH_4_	~100%	12	0.045	^6^
		TiO2 /ZnO heterojunctions	Photodegradation	UV Lamp	97%	30	—	^51^
		Au-Ag NPs	Reduction	NaBH_4_	77.57%	5	0.269	Present work
	In ternary mixture	Au-Ag NPs	Reduction	NaBH_4_	80.06%	5	0.361	Present work

### Reusability of Au-Ag catalyst

**Figure 6:**
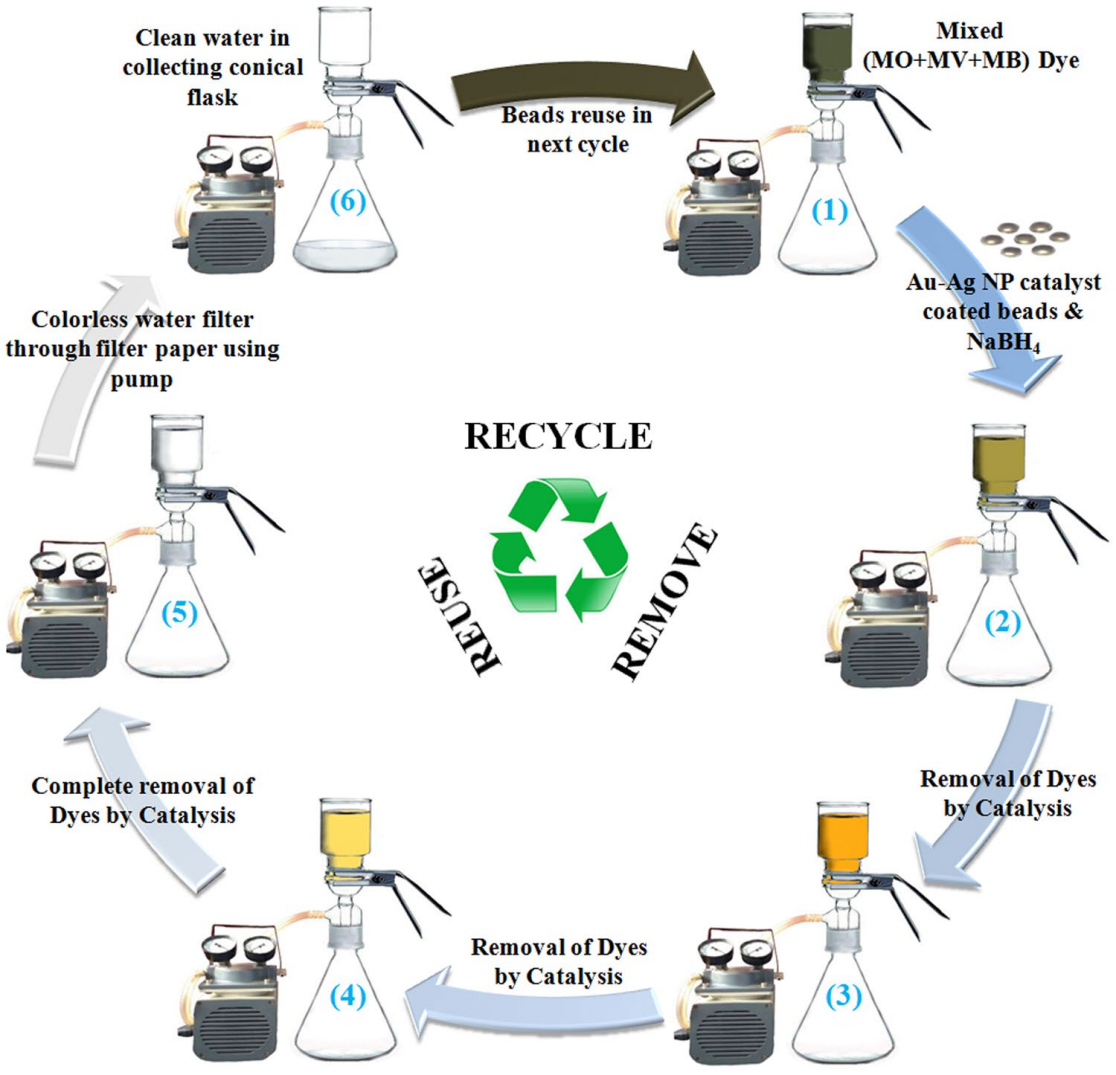
Schematic recycle model for removal of ternary mix dyes by NaBH_4_ using Au-Ag nanoparticle (NP) catalysts coated beads. The initially greenish-black mix dye solution fades gradually, as a function of the degradation progression, to green, orange, yellow and finally colorless indicative of the complete (almost) removal of dyes. Colorless liquid is filtered through 0.2 μm nylon filter paper into the conical flask on applying vacuum, after that catalyst reused upto three cycles.

**Figure 7:**
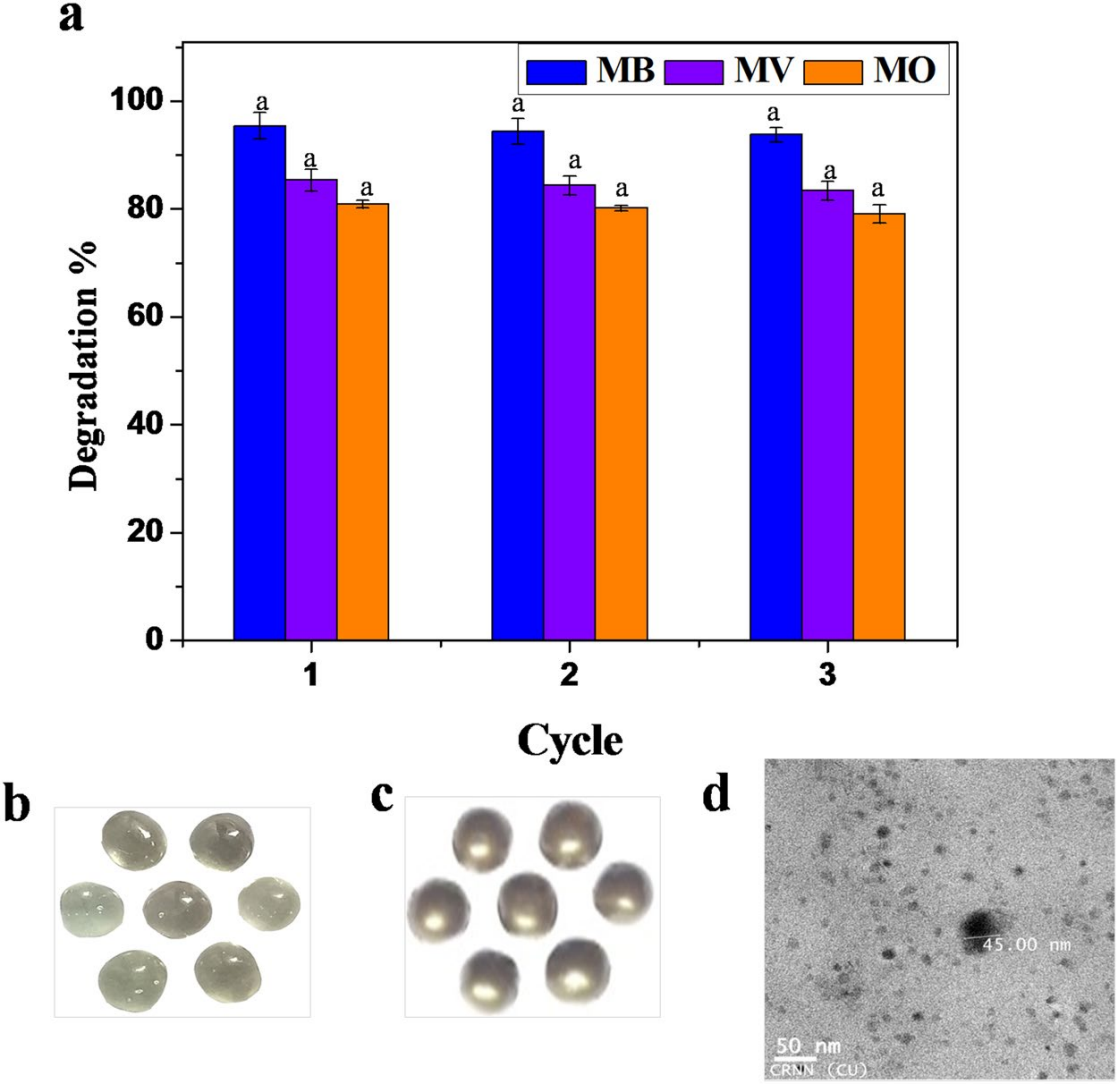
(**a**) The recycle efficiency of Au-Ag alloy NP coated catalysts for degradation of methylene blue (MB), methyl violet (MV) and methyl orange (MO) in ternary mixed aqueous solution. Photograph showing glass beads (**b**) before and (**c**) after coating with Au-Ag alloy NPs (mixture of alloy NPs with xanthan gums), (**d**) TEM image shows the uniform distribution of alloy NPs throughout the xanthan gums matrix. Data were represented as mean ± standard deviation and any two means on the same color column bars with the same letter are not significantly (*p* > 0.05) different from Duncan’s multiple range tests.

Due to certain constraints like time consuming separation, washing and drying, etc. recycling of most of the catalysts were not simple and realistic in real application methods^51–53^. The potential of Au-Ag catalyst for removing dyes in aqueous solution was, therefore, tested for reusability for practical application. Recycling of the catalyst was easy in the borosilicate glass vacuum filtration unit (Fig. 6) using Au-Ag alloy catalyst coated glass beads (Fig. 7c). TEM image of alloy coating indicated that uniform distribution of alloy NPs throughout the prepared matrix (Fig. 7d). The catalyst coated beads remained in funnel just after the colorless liquid was filtered into the conical flask at the end of reaction. The catalytic reaction cycle for degradation of dyes is schematically presented in Fig. 6. The Au-Ag catalyst could be reused for three cycles for degradation of mixed dyes in aqueous solution without significant decrease (<2–3% for each dye) of catalytic activity (Fig. 7a and UV-vis spectra shows in Fig. S14). The insignificant decrease in the chemo-catalytic efficiency might be due to their small mass losses into the reaction solution during handling in each cycling process. So, the Au-Ag NP catalyst coated beads has good reusability with high activity which makes it suitable for practical application. Therefore, the glass vacuum filtration unit using Au-Ag catalyst coated beads might be suitable for industrial application for removal of dyes from real wastewater.

## Conclusion

An easy, eco-friendly and economical strategy for biosynthesis of monometallic Au, Ag and bimetallic Au–Ag alloy NP catalysts using aqueous extract of *Polyalthia longifolia* leaf as reducing and stabilizing agent. The physico-chemical conditions were optimized for synthesis of NPs. The catalysts were characterized by various spectroscopic, microscopic, and chromatographic techniques (mostly spherical in shape, diameter 5–20 nm with prolonged stability). The catalytic activities of the synthesized NP catalysts were tested for degradation of three representative organic dyes (anionic MO and cationic MV and MB) in solution (individually and in ternary mixture). The bimetallic Au–Ag alloy NP catalysts exhibited excellent activity for degradation of ternary dyes (80.06–96.59% degradation within 5 min, k = 0.361–1.518 min^−1^, T_50_ = 0.457–1.920 min and T_80_ = 1.060–4.458 min) compared to the monometallic catalysts which motivated us to design a vacuum filtration unit using alloy catalyst coated beads for recycling. The alloy catalyst coated beads could be easily recovered and reused upto three cycles, which is important for use in real wastewater treatment. To the best of our knowledge, the bimetallic Au–Ag alloy NPs catalyst is the first example displaying significant efficiency towards the degradation of ternary dyes. The present work provides the optimized conditions for synthesis of bimetallic Au–Ag alloy NP catalysts with high stability and excellent catalytic efficiency. In addition, Au-Ag alloy NP catalysts coated glass beads offered good recycling capacity for application in industrial-scale wastewater treatment and allied areas.

## Materials and Methods

### Materials

Silver nitrate (AgNO_3_, GR 99.9%), Sodium borohydride (NaBH_4_, GR 96%), methyl orange (MO), methylene blue (MB) were purchased from MERCK (India) and Chloroauric acid (HAuCl_4_.3H_2_O, GR ~ 49%), methyl violet (MV) were procured from SRL (India) and Xanthan gum was obtained from Loba Chemie Pvt. Ltd. (India). All the chemicals were used without any further purification. Milli-Q water and borosil/borosilicate glassware were used throughout the study.

### Collection, processing, and extraction of plant

Fresh *Polyalthia longifolia* (Sonn.) Thwaites leaves were collected from the campus of Bidhan Chandra Krishi Viswavidyalaya (Mohanpur, Nadia, West Bengal, India) and washed several times with Milli-Q water and then dried under shade at room temperature. The aqueous extract of powdered *P. longifolia* leaves (10 g) was prepared by boiling with 200 ml of Milli-Q water in 500 ml Erlenmeyer flask for 30 min. The supernatant was filtered through Whatman (No. 40) filter paper and the filtrate was preserved under 4 °C for further experiment.

### Biosynthesis of catalysts

For synthesis of Ag, Au and Au–Ag NP catalysts, aqueous leaf extract of *P. longifolia* (4 ml) was added to 16 ml aqueous salt solution (1 mM) of silver nitrate, chloroauric acid, and silver nitrate/chloroauric acid (1:1) respectively. Subsequently, this setup was kept in the dark condition and heated to 70 °C for 1 h at pH–6 in case of AuNPs and pH–10 for Ag and Au–Ag NPs. After 20 min, the primary indication of the synthesis of Ag, Au, and Ag–Au alloy NPs was detected by the color change of the solution from pale yellow to golden yellow, dark-purple and reddish brown respectively. The formation kinetics of NPs was determined by measuring the absorbance of the resulting solution spectrophotometrically.

### Process optimization for biosynthesis

To evaluate the optimum condition responsible for better synthesis of AuNPs, AgNPs and Au–Ag (1:1) NPs, five different physico-Chemical conditions were studied in dark such as concentration of plant extract, concentration of the precursor salt(s), temperature, time period and various pH conditions^54^. Bimetallic Au–Ag NPs were synthesized by changing the molar ratio of the precursors (Au:Ag ratios were 0.75:0.25, 0.50:0.50 and 0.25:0.75) to investigate the effect of the ratio of the precursor salts at pH 12.0 and heated to 70 °C temperature. AgNPs and AuNPs were synthesised following the same method for comparison. The effect of these parameters on the formation of bimetallic NPs was observed by UV–vis spectrophotometer. The biosynthesized NPs were separated by centrifugation (14,000 rpm for 15 min), washed several times with Milli-Q water. Finally, the centrifuged products air dried and used for further studies.

### Characterization of the catalysts

The optical absorption spectra of synthesized NPs were recorded by using a UV–vis spectrophotometer (Varian, Cary 50) at room temperature. Morphology and structure of the synthesized NPs was studied using Transform electron microscopy (TEM; JEOL, JEM–2100) operating at 200 kV of acceleration voltage equipped with Energy dispersive X–ray Spectroscopy (EDX; Oxford Instruments, UK) detector to examine the elemental composition of the sample. For this study, sample of the NPs were prepared onto carbon coated copper grid and dried overnight in air at room temperature. The average size, size distribution, polydispersity index and zeta potentials of the metal NPs in the experimental colloidal solution were determined by Dynamic light scattering (DLS; Malvern, Nano-ZS) instrument using clear disposable cuvettes. The polydispersity index (PDI) indicates homogeneity of size and zeta (ζ) potential provides an idea pertaining to the stability of synthesized NPs.

Fourier transform infrared spectroscopy (FTIR; Perkin Elmer L 120–000 A) was done to identify the possible functional groups responsible for the reduction and stabilization of the NPs. All measurements were read in the wavelength between 4000–450 cm^−1^ with a resolution of 4.0 cm^−1^. For FTIR analysis, the samples of plant extract before and after formation of NPs was dried in a hot air oven dryer at 60 °C temperature and potassium bromide was added to dried sample (100:1).

The chemical constituents in plant extract before and after formation of NPs were analysed using a Gas Chromatograph (Agilent Technologies 7890 A) coupled to a Mass Spectrometric system (7000, GC/MS Triple Quad). Ionization of the sample compounds was performed in full scan mode (m/z 35 to 350) using positive electron ionization (+EI) with electron energy of −70 eV. The identification of compounds was conducted by comparing mass spectra with the National Institute of Standards and Technology (NIST MS Search 2.0) mass spectral library.

### Catalytic activity test

The chemo-catalytic activities of the synthesized NPs were evaluated by measuring the degradation of MB or MV or MO and ternary mixed dye aqueous (Milli-Q water) solution, of two cationic dyes (MB and MV) and an anionic dye (MO). In a 3 ml of quartz cuvette, 50 μl of MB or MV or MO dye solution (1 mM) was individually mixed with 50 μl of freshly prepared NaBH_4_ (0.2 M) solution and 50 μl of catalyst (1 mM) solution. Similarly, for degradation of ternary mixed dye solution, 1 mM of MB, MV and MO (50 μl each) dye solution was mixed with 150 μl freshly prepared NaBH_4_ (0.2 M) aqueous solution and 50 μl of catalyst (1 mM). Control experiments were carried out without the addition of catalyst. Total volume of the reaction mixture was made up to 3 ml by adding Milli-Q water and kept at constant room temperature (25 °C) and at natural pH to eliminate the thermal and pH effect on the process of catalysis. The concentration of dye solution wascontinuously monitored by measuring their respective λ_max_ values using a UV–vis spectrophotometer (Varian, Cary 50) at specific intervals of time in the range of 200–800 nm at room temperature.

The chemo-catalytic performance was determined with the following formula:

1 $${\rm{Percentage}}\,{\rm{degradation}}=\frac{({{\rm{A}}}_{0}-{{\rm{A}}}_{{\rm{t}}})}{{{\rm{A}}}_{0}}\times 100$$

where, A_0_ is the initial absorbance of dye solution; A_t_ is the absorbance of dye solution over selected time intervals.

The reaction kinetics of dye degradation was usually determined by modified first-order kinetics law and it may be expressed as:

2 $${\rm{In}}\,\frac{{{\rm{C}}}_{{\rm{t}}}}{{{\rm{C}}}_{0}}=-\,{\rm{kt}}$$

where t is the reaction time, k is the apparent first-order rate constant. C_0_ is the initial absorbance of dye solution and C_t_ is the concentration of dye solution during reaction at a specific time (t). The apparent first-order rate constant (k) was determined from the slope ln(C_t_/C_0_) *versus* reaction time (t). The linear regression coefficient (R^2^) values used to represent the similarity between the experimental data and first order kinetics equation.

To compare the performance of catalytic activity of the NPs, half-life (T_50_) and time required for 80% degradation (T_80_) of the dyes was calculated using the following formula:

3 $${{\rm{T}}}_{50}=\frac{\mathrm{ln}\,2}{{\rm{k}}}=\frac{0.693}{{\rm{k}}}$$

4 $${{\rm{T}}}_{80}=\frac{\mathrm{ln}\,5}{{\rm{k}}}=\frac{1.609}{{\rm{k}}}$$

### Method for reuse of catalyst

A practical method for removal of mixed dye was standardized using borosilicate glass vacuum filtration (Borosil 5350030) unit with catalyst coated borosilicate glass bead. The device consisted of a borosilicate glass funnel (300 ml) with filter holder, spring clamp, filter flasks (2 L) and membrane filter paper (Pall Life Sciences Ultipor N66, Nylon 6,6 membrane, 47 mm). A constant flow pump (Millipore XI0422050) was connected to the filter holder to adjust the flow rate.

Au-Ag alloy NPs suspension (2%) and xanthan gum (0.5%) in water (10 ml) was thoroughly mixed and added to borosilicate glass beads (200 nos. with ~2 mm diameter) for coating of NPs onto the glass beads and dried at 120 °C for 2 h. The catalytic degradation of ternary mixture of dyes (1 mM, 4 ml each of MB, MV and MO) was carried out in glass funnel (borosilicate, 300 ml) at room temperature after addition of water (216 ml) followed by addition of glass beads (20 nos.) coated with Au-Ag alloy catalyst. Freshly prepared aqueous solution of NaBH_4_ (0.2 M, 12 ml) was then added into the funnel for degradation of dyes. At the end of the catalytic reaction, the colorless liquid was filtered through 0.2 μm nylon filter paper into the conical flask under vacuum (approx. 125 mm Hg). The catalyst coated glass beads were further used up to three cycles following vacuum filtration and evaluated for catalytic activity after each cycle.

### Statistical analysis

All the batch experiments were performed at least in triplicate and data were presented as mean ± standard deviations in this study. Statistical analyses were carried out using the Statistical Package for Social Sciences (SPSS) version 25.0. One-way analysis of variance (ANOVA) was performed using the SPSS program and the significance of each mean value was determined (*p* < 0.05) by Duncan’s multiple range test.

## Supplementary information

Supplementary Information.
