# Author Correction: Process optimization for biosynthesis of mono and bimetallic alloy nanoparticle catalysts for degradation of dyes in individual and ternary mixture

**DOI:** 10.1038/s41598-020-62930-y

**Published:** 2020-04-03

**Authors:** Sabyasachi Ghosh, Swarup Roy, Jishu Naskar, Ramen Kumar Kole

**Affiliations:** 1 Department of Biochemistry and Biophysics, University of Kalyani, Kalyani, Nadia, 741235 West Bengal India; 2 BioNanocomposite Research Center, Department of Food and Nutrition, Kyung Hee University, 26 Kyungheedae–ro, Dongdaemun–gu, Seoul 02447 Republic of Korea; 3 Department of Agricultural Chemicals, Bidhan Chandra Krishi Viswavidyalaya, Mohanpur, Nadia, 741252 West Bengal India

Correction to: *Scientific Reports* 10.1038/s41598-019-57097-0, published online 14 January 2020

The Supplementary Information file that accompanies this Article contains an error where Figure S1 is a duplication of Figure S2. The correct Figure S1 appears below as Figure 1.

**Figure 1:**
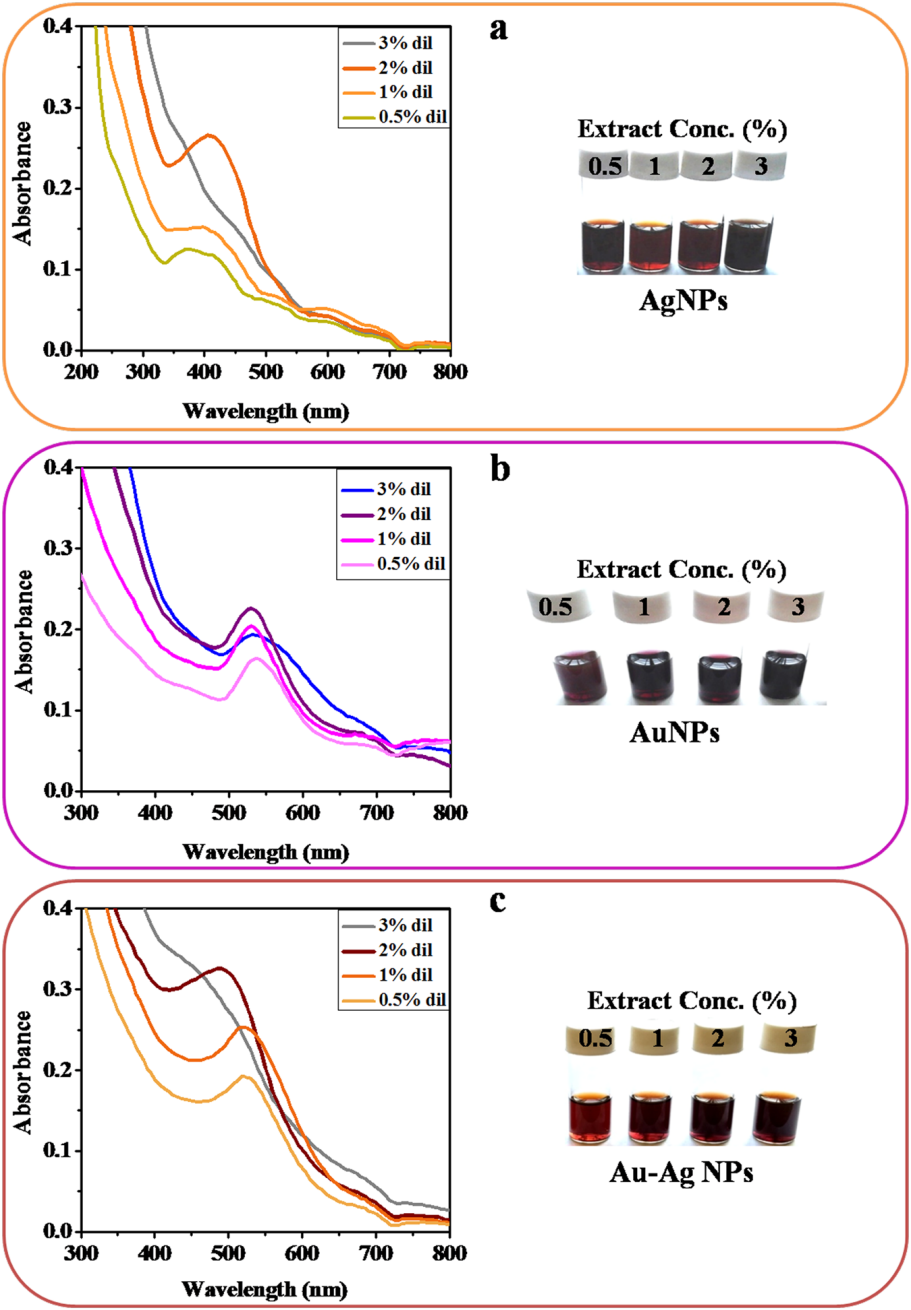
Plant extract-dependent UV–vis absorbance and corresponding visual color change of the biosynthesized (**a**) AgNPs, (**b**) AuNPs and (**c**) Ag-Au alloy NPs.

