# Supplementary material for: Process optimization for biosynthesis of mono and bimetallic alloy nanoparticle catalysts for degradation of dyes in individual and ternary mixture
Source: Sci Rep. 2020 Jan 14;10:277. doi: 10.1038/s41598-019-57097-0 (PMC6959256; doi:10.1038/s41598-019-57097-0)
Supplement: Supplementary file 1 — Supplementary Information. [file 41598_2019_57097_MOESM1_ESM.docx]

**Supplementary Information**

**Process optimization for biosynthesis of mono and bimetallic alloy nanoparticle catalysts for degradation of dyes in individual and ternary mixture**

Sabyasachi Ghosh^1,3^, Swarup Roy^2^, Jishu Naskar^1^ and Ramen Kumar Kole^3^*

^1^Department of Biochemistry and Biophysics, University of Kalyani, Kalyani, Nadia–741235, West Bengal, India.

^2^BioNanocomposite Research Center, Department of Food and Nutrition, Kyung Hee University, 26 Kyungheedae–ro, Dongdaemun–gu, Seoul 02447, Republic of Korea.

^3^Department of Agricultural Chemicals, Bidhan Chandra Krishi Viswavidyalaya, Mohanpur, Nadia–741252, West Bengal, India.

*Corresponding author.

*E–mail address*: [rkkole@yahoo.com](mailto:rkkole@yahoo.com)

**Table of Contents**

1. **Supplementary Figures**
2. **Supplementary Tables**
3. **Supplementary Notes**
4. **Supplementary References**
5. **Supplementary Figures**


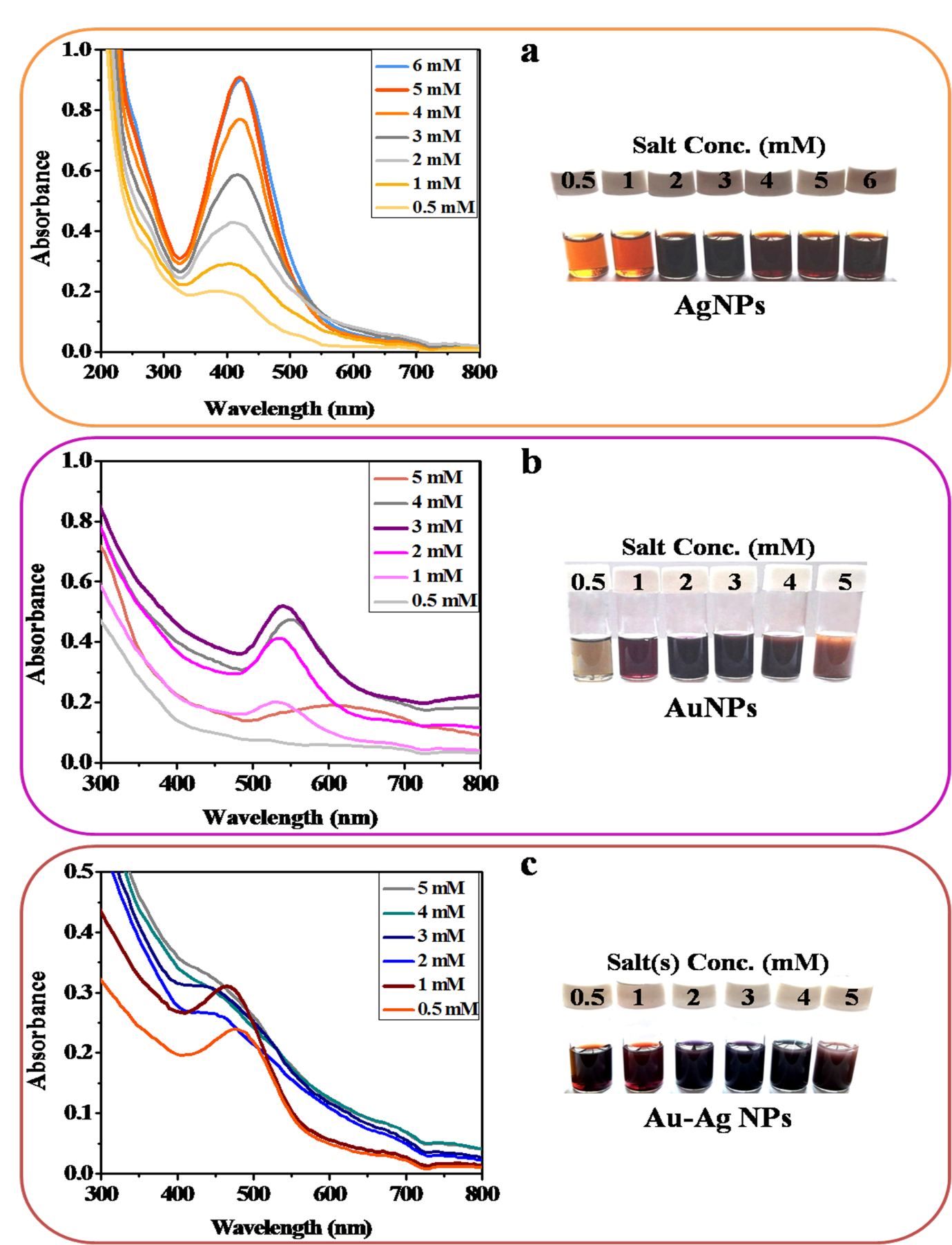


**Figure S1.** Plant extract-dependent UV–vis absorbance and corresponding visual color change of the biosynthesized (**a**) AgNPs, (**b**) AuNPs and (**c**) Ag-Au alloy NPs.

**
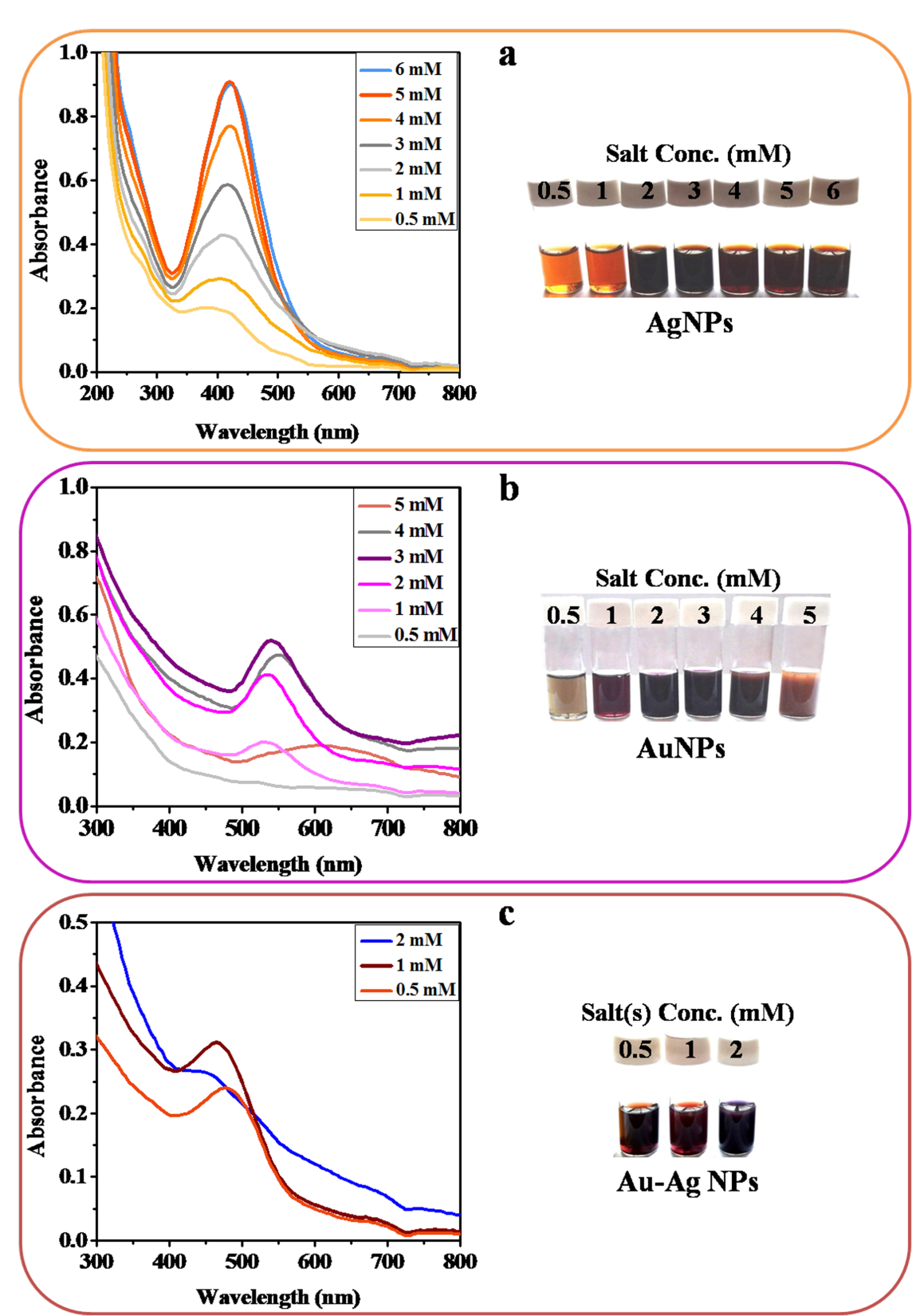
**

**Figure S2.** Precursor salt(s)-dependent UV–vis absorbance and corresponding visual color change of the biosynthesized (**a**) AgNPs, (**b**) AuNPs and (**c**) Ag-Au alloy NPs.

**
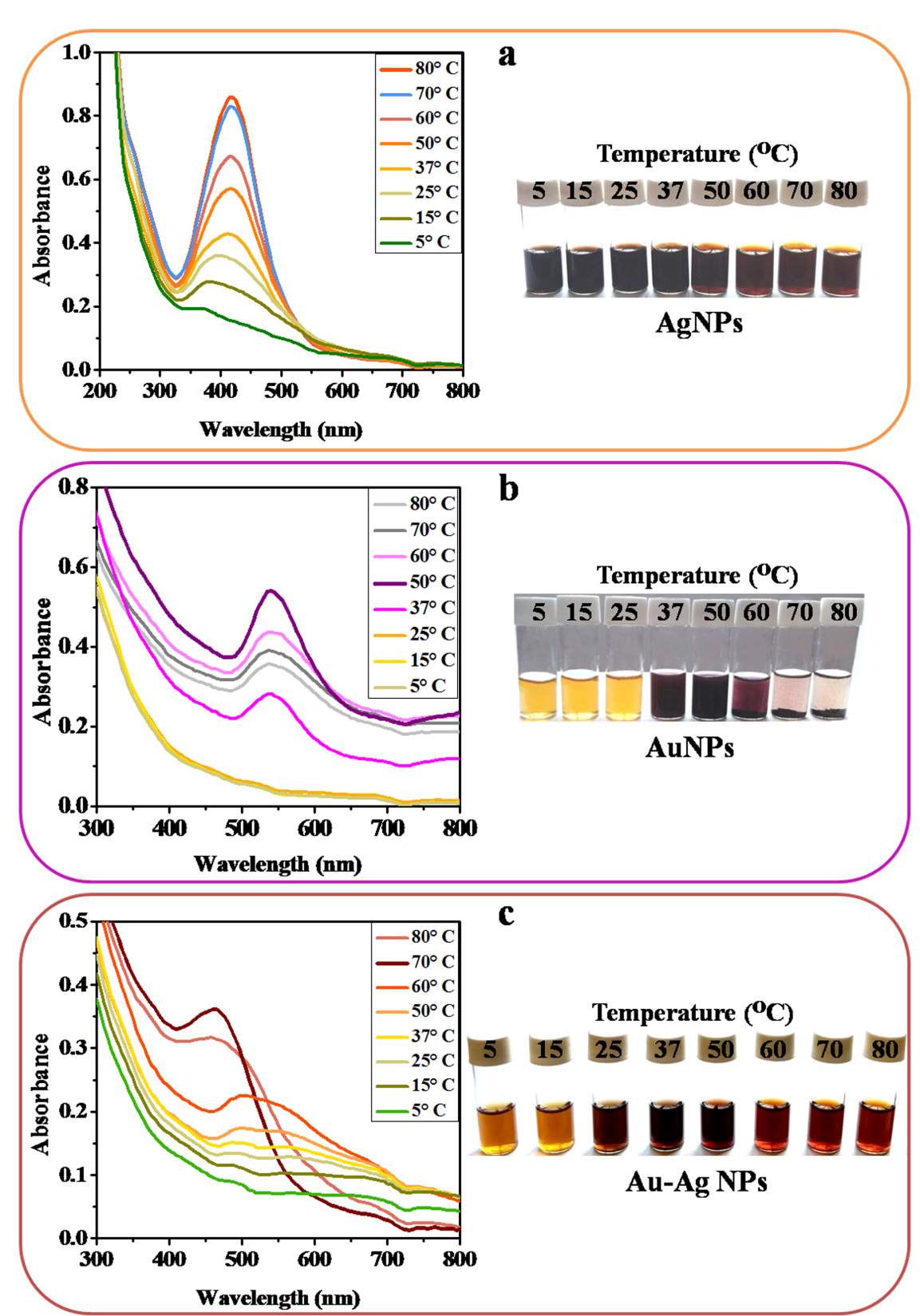
**

**Figure S3.** Temperature-dependent UV–vis absorbance and corresponding visual color change of the biosynthesized (**a**) AgNPs, (**b**) AuNPs and (**c**) Ag-Au alloy NPs.

**
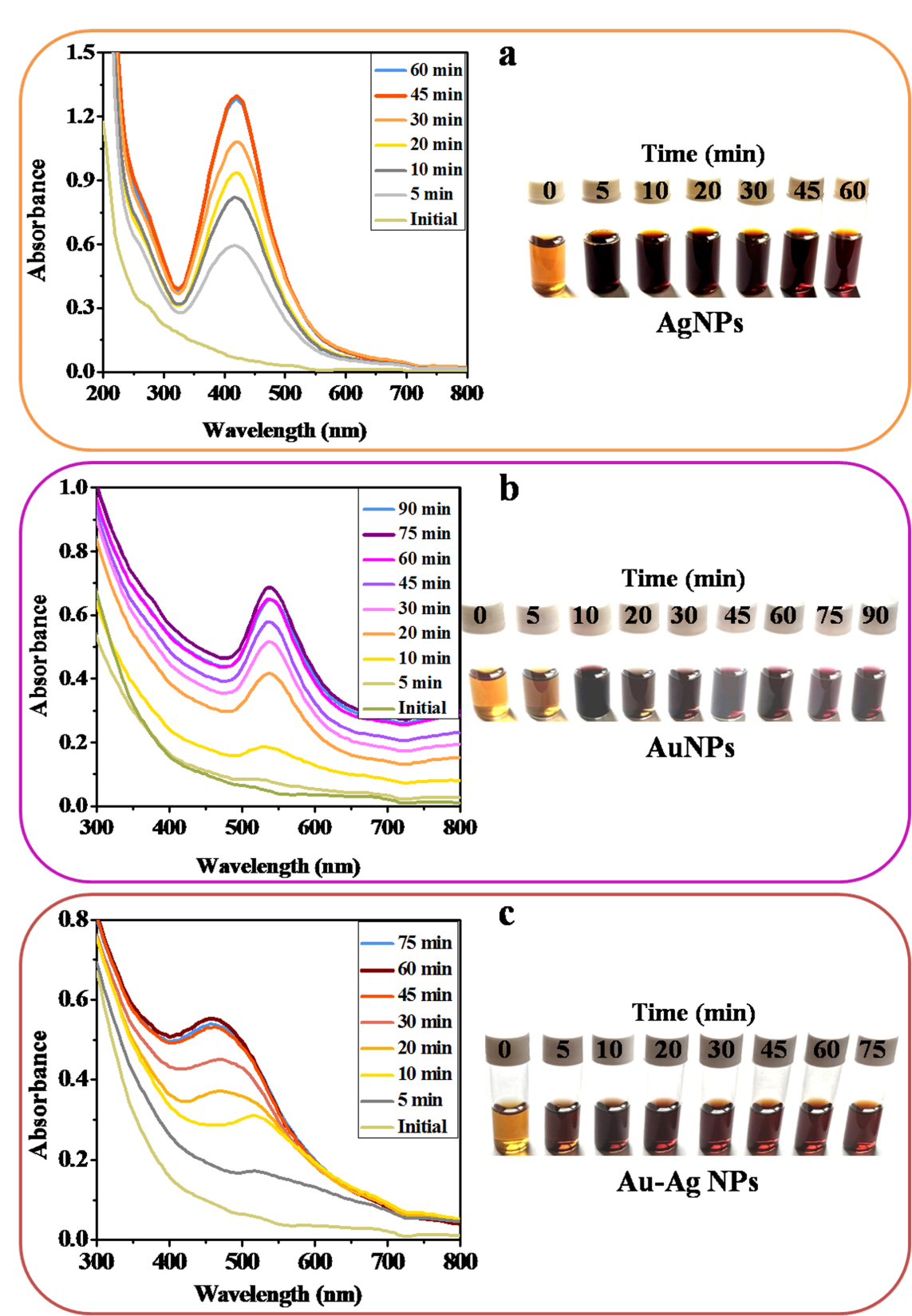
**

**Figure S4.** Time-dependent UV–vis absorbance and corresponding visual color change of the biosynthesized (**a**) AgNPs, (**b**) AuNPs and (**c**) Ag-Au alloy NPs.

**
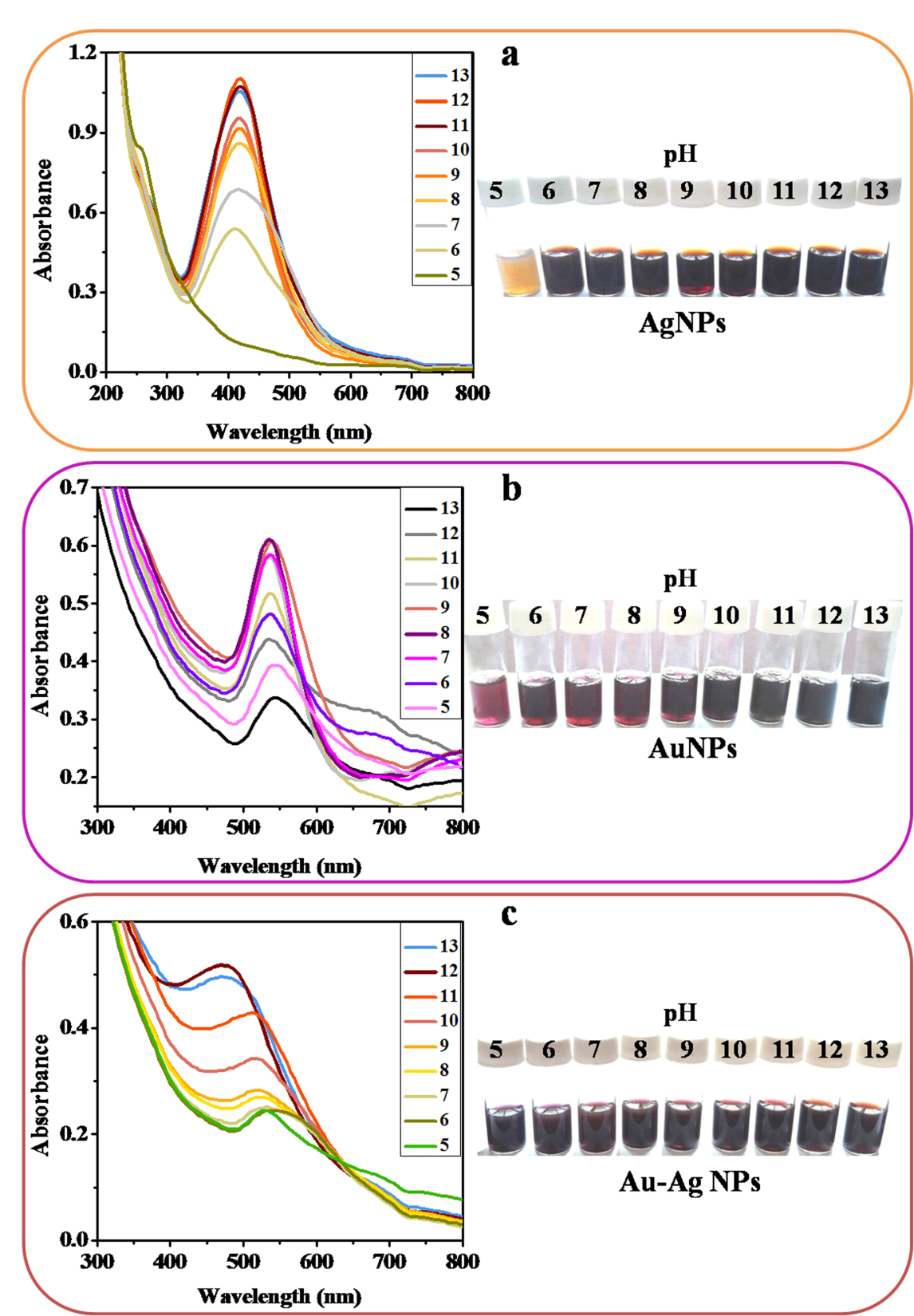
**

**Figure S5.** pH-dependent UV–vis absorbance and corresponding visual color change of the biosynthesized (**a**) AgNPs, (**b**) AuNPs and (**c**) Ag-Au alloy NPs.

**
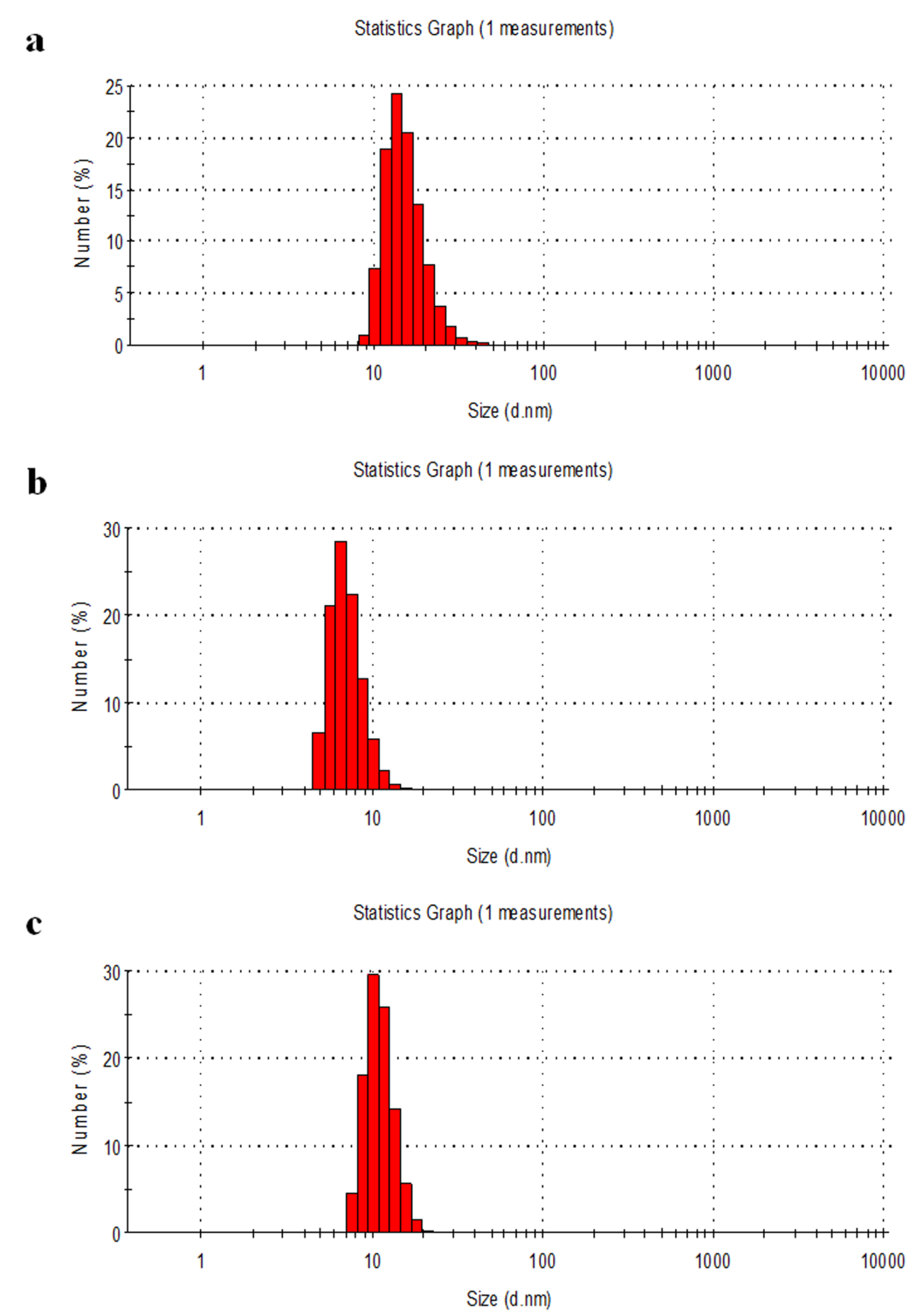
**

**Figure S6.** Size distribution histograms of the biosynthesized (**a**) AgNPs, (**b**) AuNPs, and (**c**) Au-Ag alloy NPs.

**
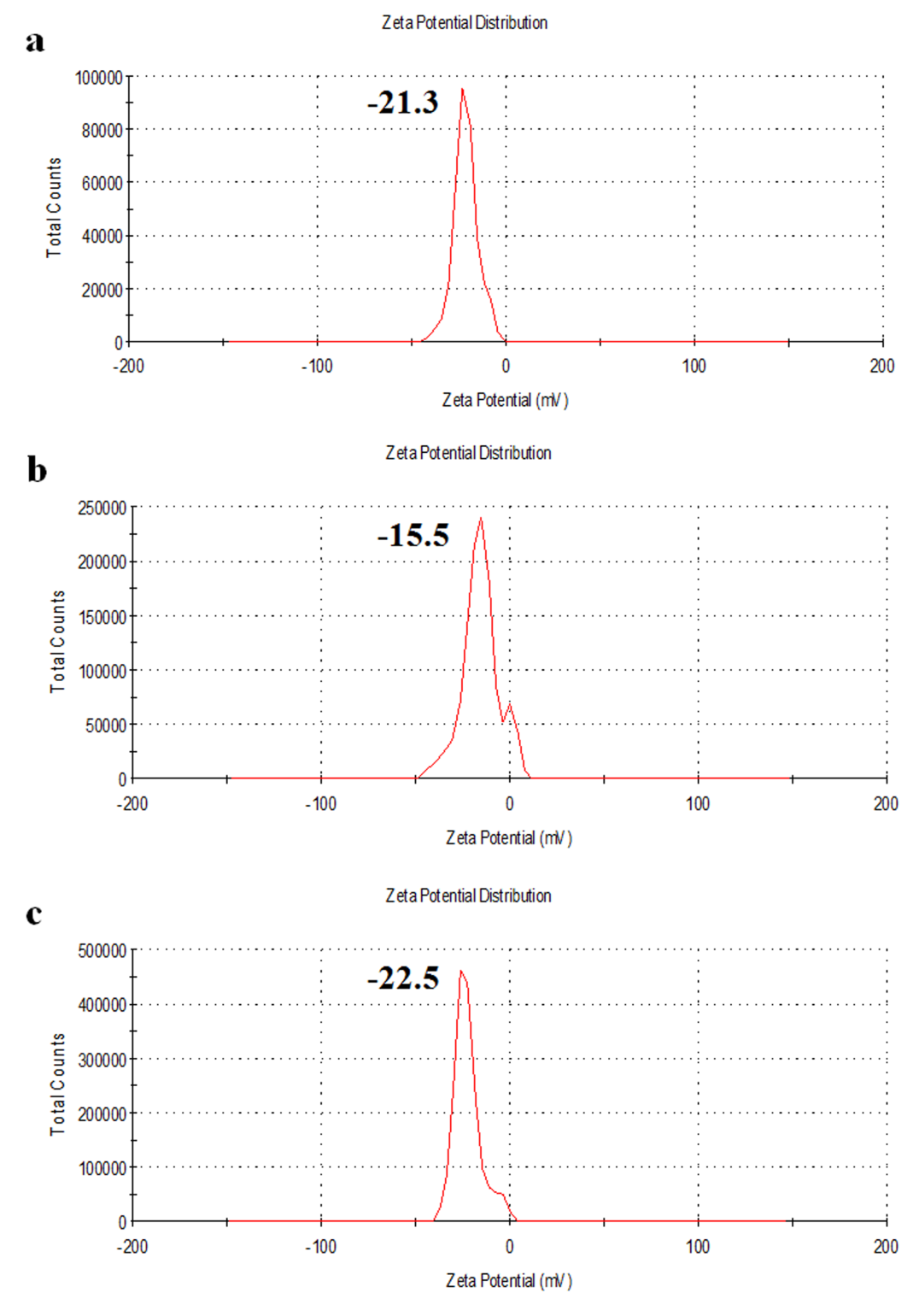
**

**Figure S7.** Zeta potential of the biosynthesized (**a**) AgNPs, (**b**) AuNPs, and (**c**) Au-Ag alloy NPs.


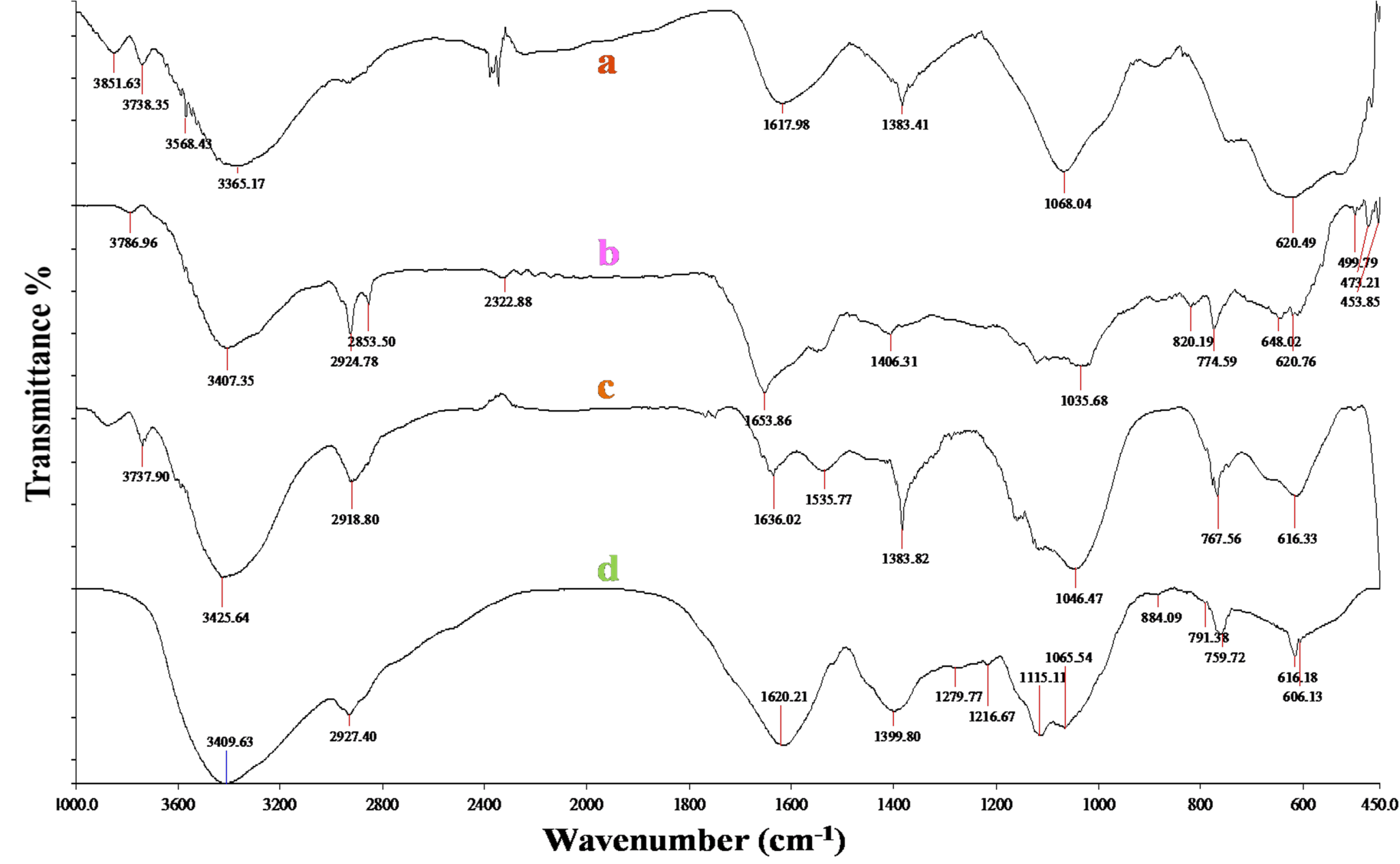
**Figure S8.** FTIR spectrum of biosynthesized (**a**) Ag-Au alloy NPs, (**b**) AuNPs, (**c**) AgNPs, and (**d**) *Polyalthia* leaf extracts.


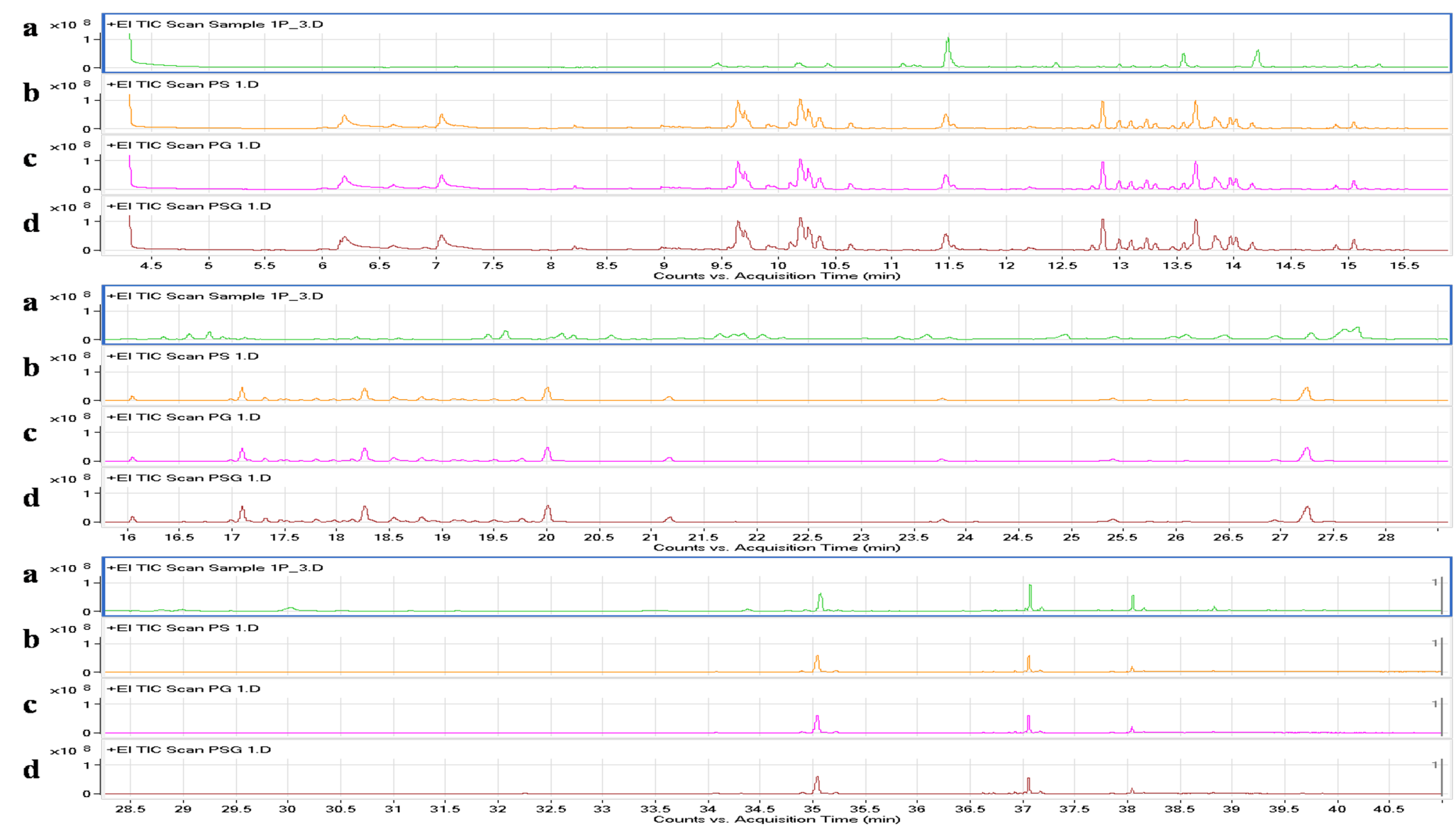


**Figure S9.** GC-MS/MS chromatogram of (**a**) *Polyalthia* leaf extracts and biosynthesized (**b**) AgNPs, (**c**) AuNPs and (**d**) Ag-Au alloy NPs.


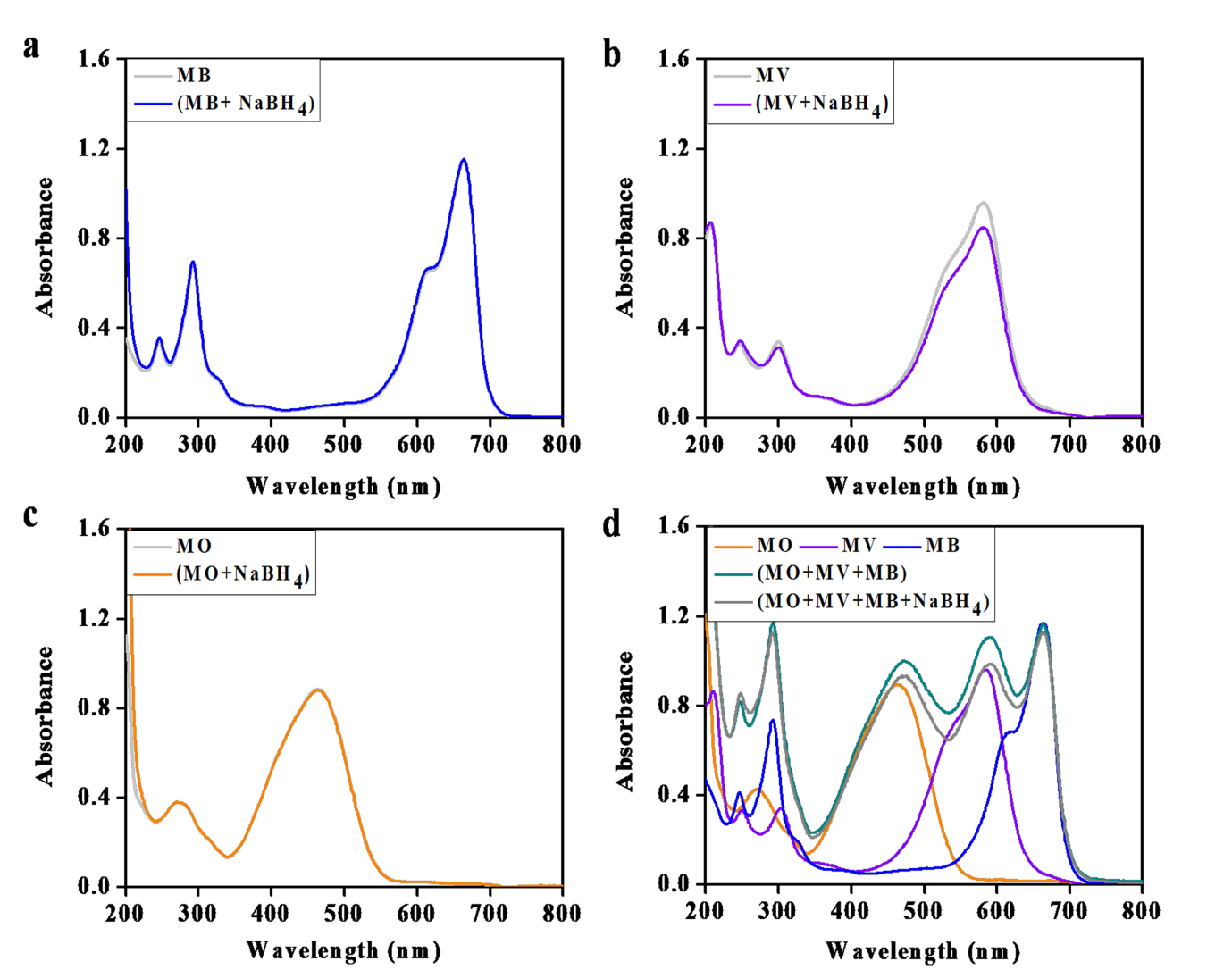


**Figure S10.** UV–vis spectra of (**a**) MB, (**b**) MV, (**c**) MO and (**d**) mixed (MO+ MV+ MB) dyes with NaBH_4_ in absence of NP as catalyst.

**
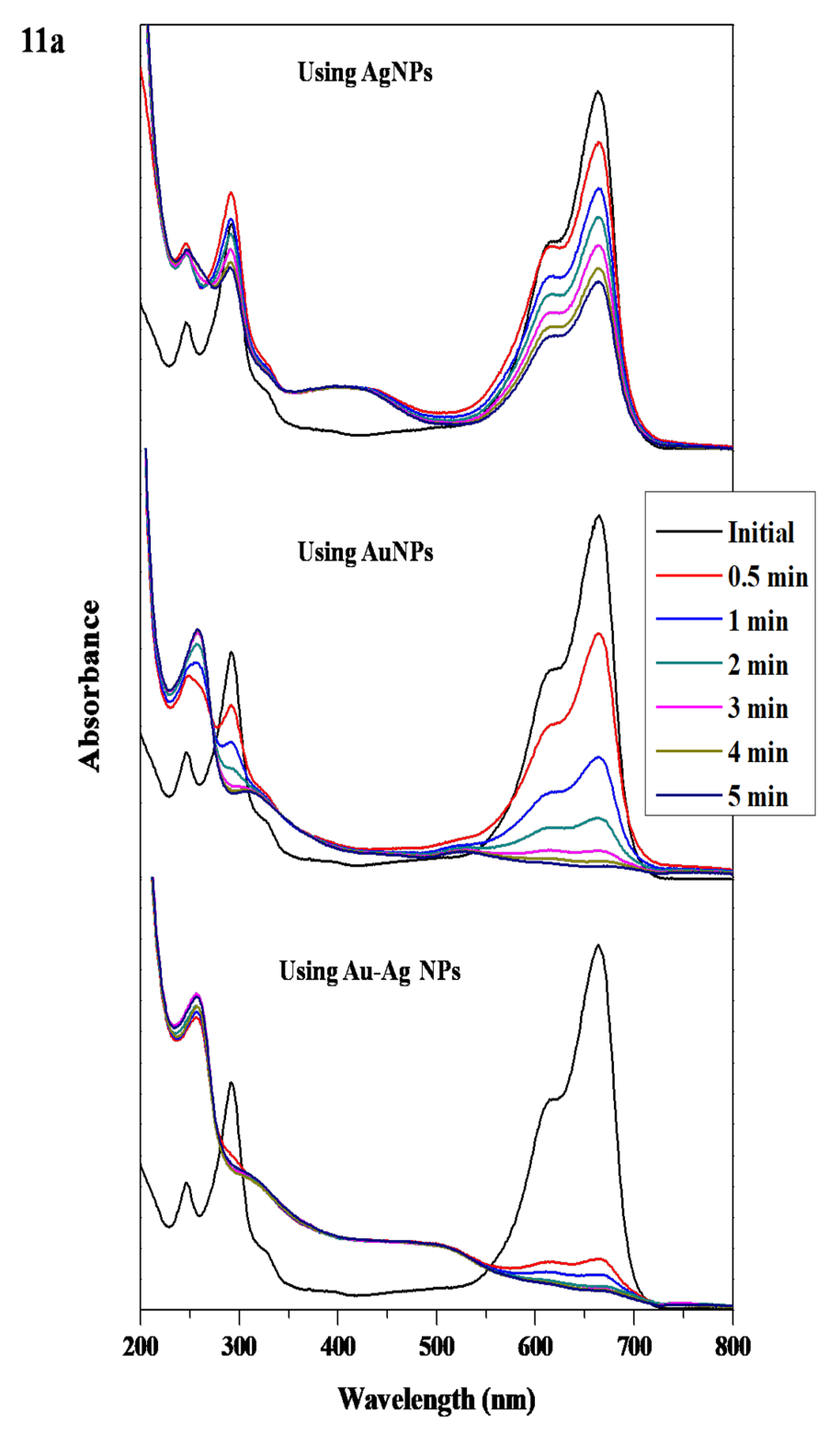
**

**Figure S11.** Time-dependent UV-vis absorption spectra for degradation of (**a**) MB, (**b**) MV and (**c**) MO dye by NaBH_4_ in presence of NPs as catalysts. Catalysts used are shown in the upper side of each degradation profile.

**
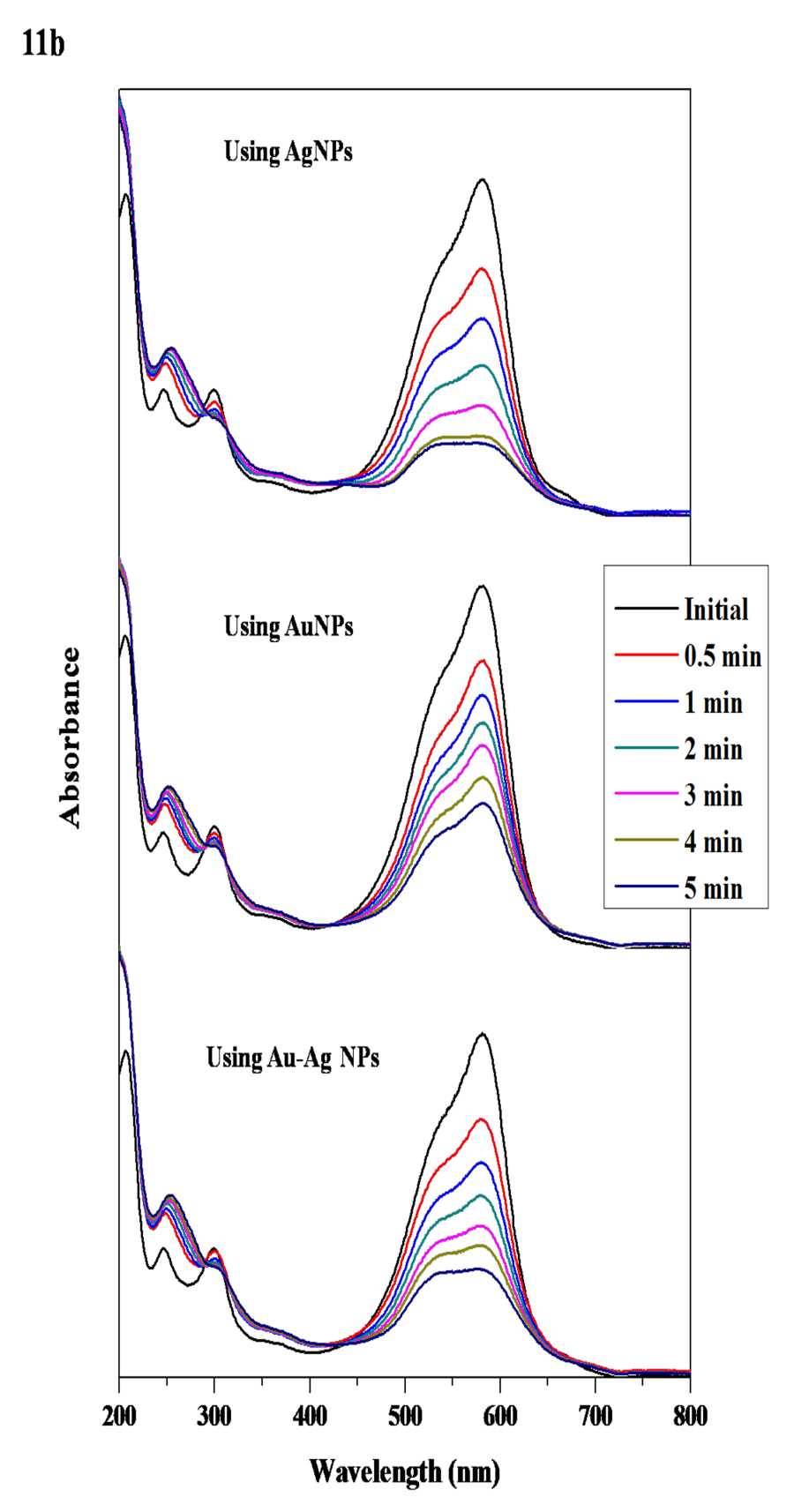
**

**
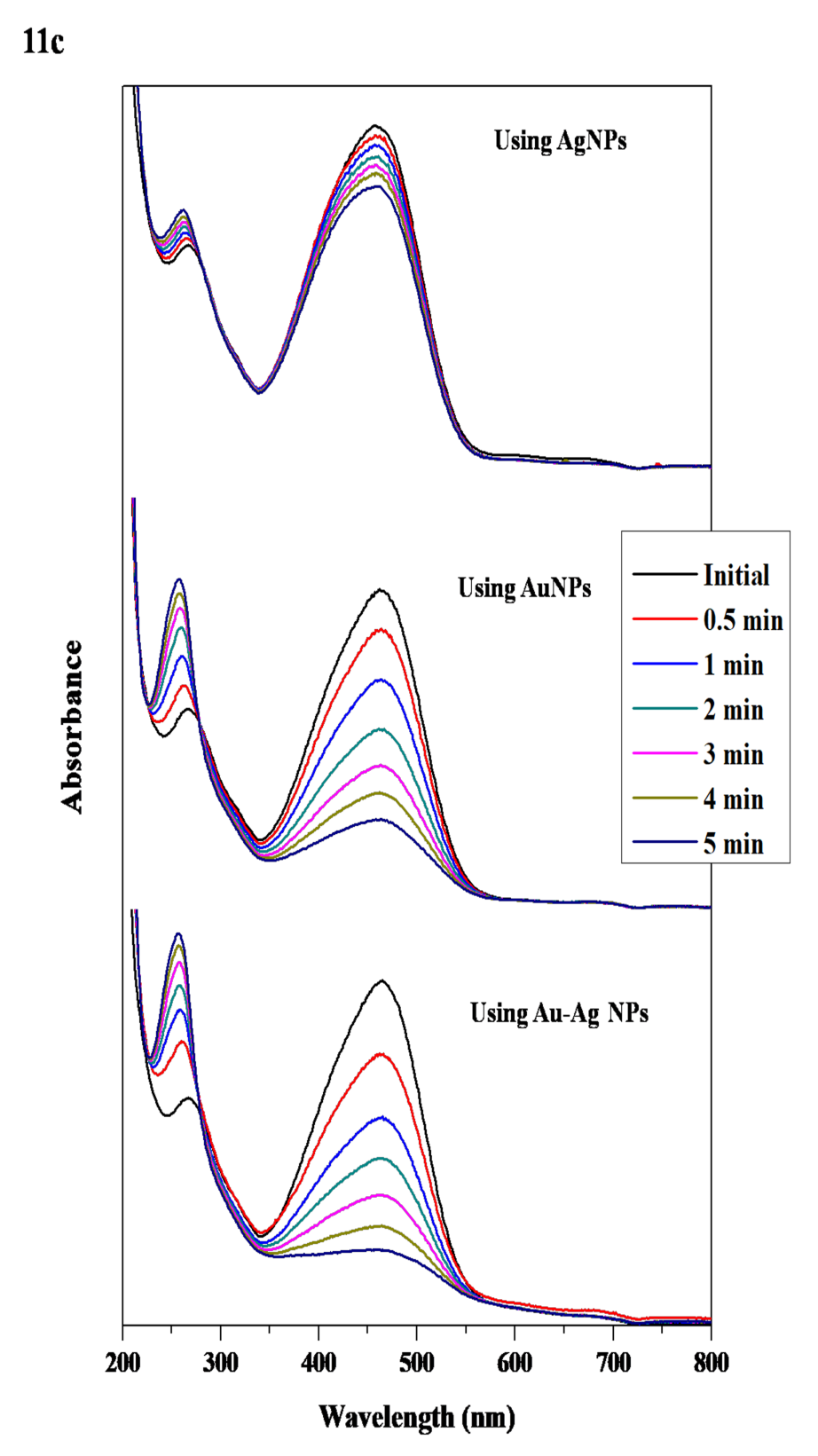
**

**
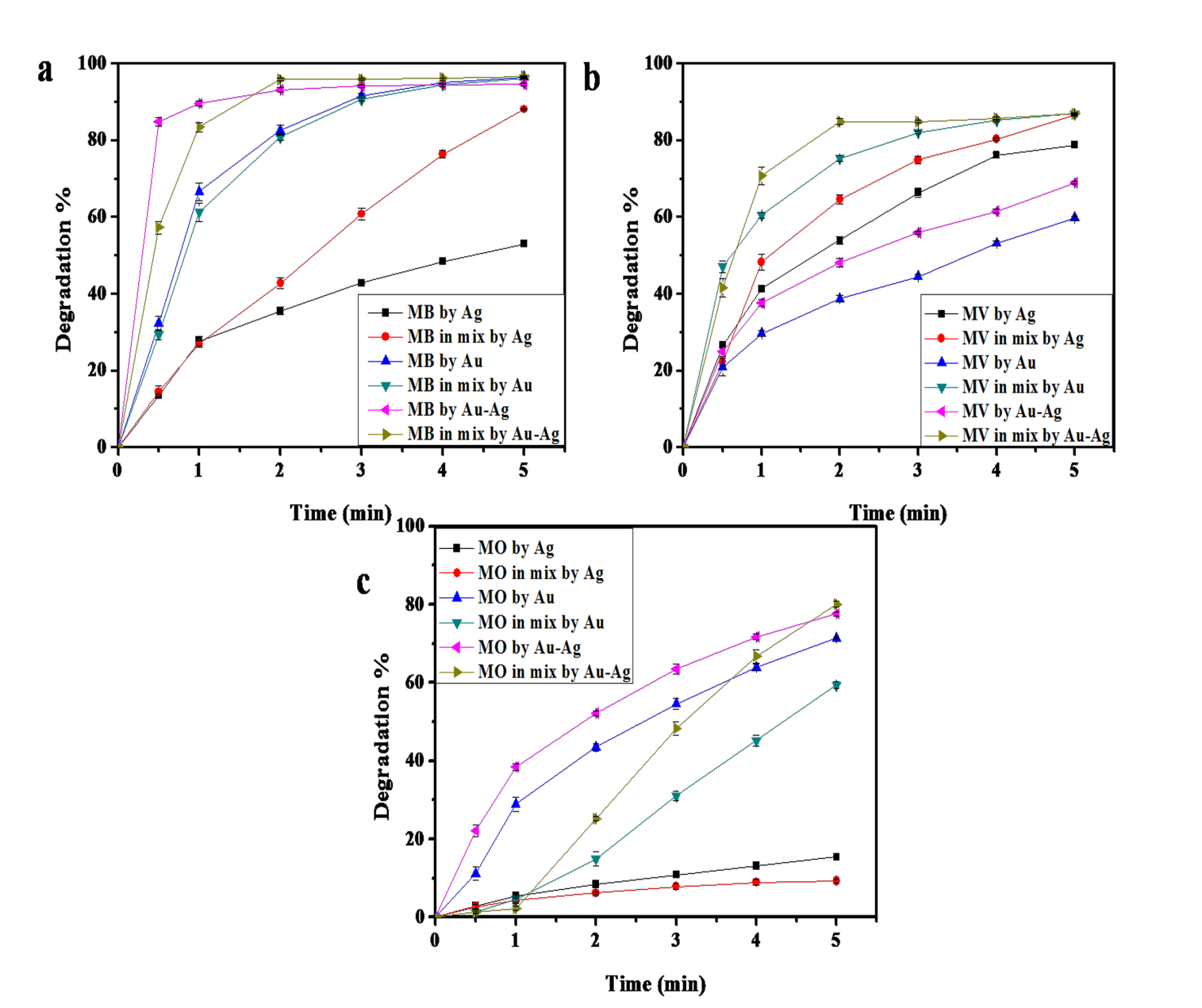
**

**Figure S12.** Time-dependent degradation percentage profile of (**a**) MB, (**b**) MV and (**c**) MO dyes in ternary mixed and individual against time by NaBH_4_ in presence of NPs as catalysts. Bars indicate standard error (±SE)

**
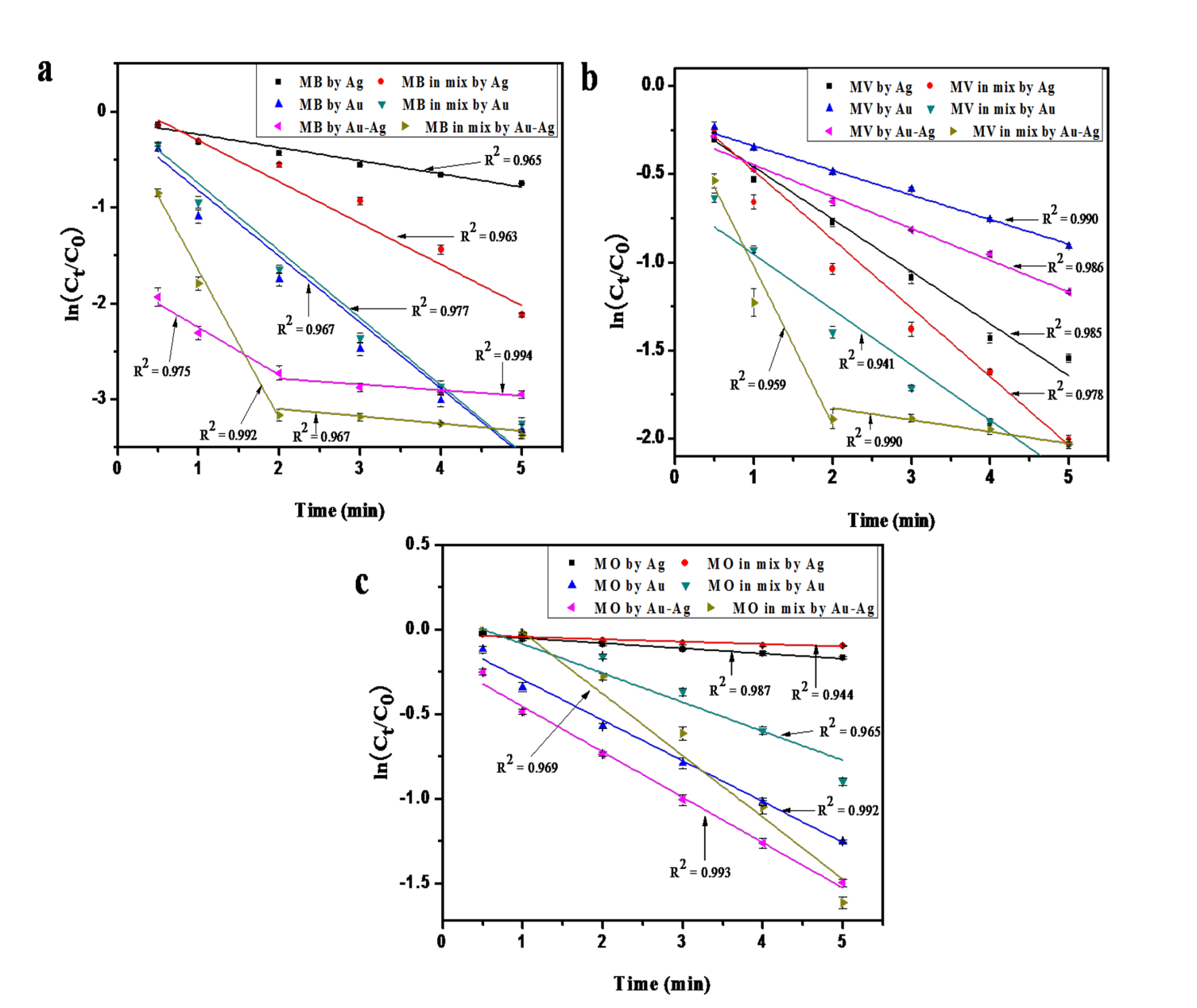
**

**Figure S13.** Degradation Kinetics of (**a**) MB, (**b**) MV and (**c**) MO dyes in ternary mixed and individual solution by NaBH_4_ in presence of NPs as catalysts. Bars indicate standard error (±SE)

**
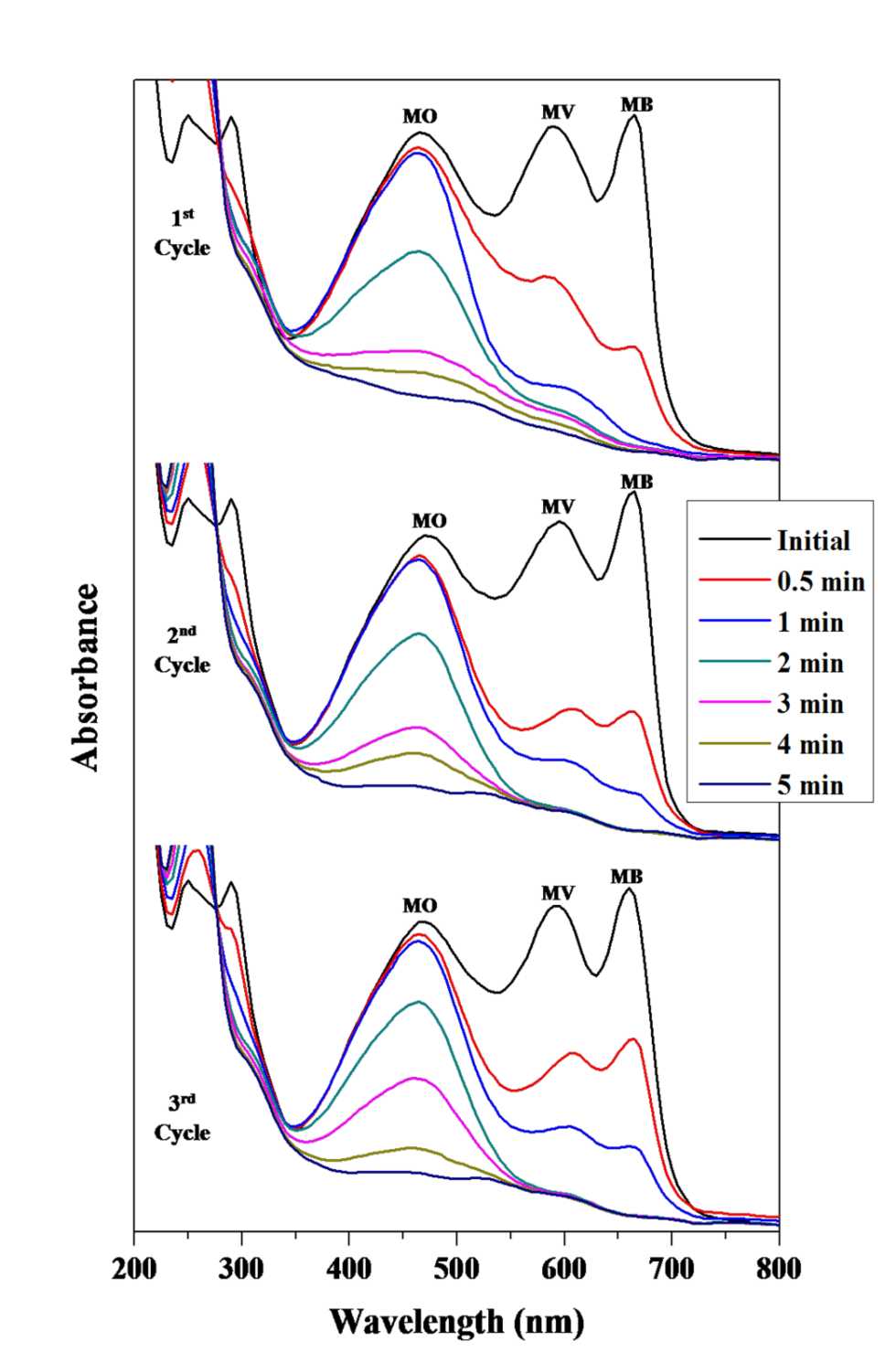
**

**Figure S14.** UV-vis spectra for degradation of ternary dyes by NaBH_4_ in presence of Au-Ag NP catalyst coated beads used in 1^st^ cycle, 2^nd^ cycle and 3^rd^ cycle.

**2. Supplementary Tables**

| **Sl. No.** | **FTIR absorption (cm^–1^)** | | | | **Assigned functional groups** |
| --- | --- | --- | --- | --- | --- |
|  | **Plant extract** | **Extract based AgNPs** | **Extract based AuNPs** | **Extract based Au–Ag NPs** |  |
|  | 3409.63 | 3425.64 | 3407.35 | 3568.43 | Stretching vibration of O–H groups, Alcohols and Phenols |
|  |  | 2918.80 | 2924.78 | 2853.50 | C–H stretching vibration, hydrocarbon part of biomolecules |
|  |  | 1636.02 | 1653.86 |  | –C=C– stretch, hydrocarbon part of biomolecules |
|  | 1620.21 |  |  | 1617.98 | bending vibration of N–H groups |
|  |  | 1535.77 |  |  | C=O stretching vibration of amides groups |
|  |  |  | 1406.31 |  | C–C Stretch, aliphatic |
|  | 1399.80 |  |  |  | bending vibration of N=O groups |
|  |  | 1383.82 |  | 1383.41 | C–C stretch , Aromatics |
|  | 1279.77, 1216.67, 1115.11 |  |  |  | C=O stretching vibration of ester and carboxylic acids |
|  | 1065.54 | 1046.47 | 1035.68 | 1068.04 | C=O stretching vibration of ester, carboxylic acids and alcoholic functional sites of biomolecules |

**Table S1.** FTIR spectra with probable corresponding functional groups present in plant extract and biosynthesized nanoparticles.

| **Sl.**  **No.** | **Retention Time (min)** | | **Compound** | **Probability of Identification** |
| --- | --- | --- | --- | --- |
|  | **Plant extract** | **Extract based NPs** |  |  |
| 1. |  | 6.19 | Heptane, 2,4-dimethyl- | 42.5 |
| 2. |  | 7.04 | Octane, 4-methyl- | 53.1 |
| 3. | 9.46 |  | Benzyl alcohol | 65.5 |
| 4. |  | 9.63 | α-Methyl-α-[4-methyl-3-pentenyl]oxiranemethanol | 36.6 |
| 5. | 10.19 | 10.19 | Phenylethyl Alcohol | 82.8 |
| 6. |  | 10.32 | Undecane | 24.2 |
|  |  | 10.43 | Undecane, 4-methyl- | 29.9 |
| 7. | 11.47 | 11.47 | Azulene | 51.3 |
| 8. |  | 12.79 | 1,4-dihydroxy-p-menth-2-ene | 53.9 |
| 9. | 12.96 | 12.96 | Hexanedioic acid, dimethyl ester | 92.2 |
| 10. | 13.06 | 13.06 | 3-Cyclohexene-1-methanol, 2-hydroxy-α,α,4-trimethyl- | 25.5 |
| 11. |  | 13.24 | 11-Methyldodecanol | 36.7 |
| 12. | 13.55 | 13.55 | 2-Methoxy-4-vinylphenol | 61.9 |
| 13. |  | 13.68 | Dodecane, 2,6,11-trimethyl- | 15.9 |
| 14. |  | 13.83 | Dodecane, 2,7,10-trimethyl- | 18.3 |
| 15. |  | 13.97 | Dodecane, 4-methyl- | 16.5 |
| 16. | 14.23 | 14.20 | 3,6-Octadien-1-ol, 3,7-dimethyl-, (Z)- | 49.5 |
| 17. | 15.05 | 15.05 | Pentanedioic acid, dimethyl ester | 91.8 |
| 18. | 16.59 |  | 1,2,3-Trimethoxybenzene | 20.4 |
| 19. | 16.78 |  | Cyclohexane-1-methanol, 3,3-dimethyl-2-(3-methyl-1,3-butadienyl)- | 18.7 |
| 20. |  | 17.09 | 1-Decanol, 2-hexyl- | 15.4 |
| 21. |  | 18.30 | Benzene, 1-(1,5-dimethyl-4-hexenyl)-4-methyl- | 87.1 |
| 22. | 19.62 |  | Ethanone, 1-(1a,2,3,5,6a,6b-hexahydro-3,3,6a-trimethyloxireno[g]benzofuran-5-yl)- | 84.4 |
| 23. |  | 20.04 | Hexadecane | 25.1 |
| 24. | 20.15 |  | 6-(3,3-Dimethyl-oxiran-2-ylidene)-5,5-dimethyl-hex-3-en-2-one | 26.3 |
| 25. |  |  | 2(3H)-Benzofuranone, hexahydro-4,4,7a-trimethyl- | 27.5 |
| 26. |  | 21.17 | Heptacosane | 19.2 |
| 27. | 25.42 | 25.42 | Decanoic acid, decyl ester | 69.7 |
| 28. | 27.28 | 27.28 | Octadecane | 20.9 |
| 29. | 27.72 |  | 2,3,3-Trimethyl-2-(4-methylpentanoyl)-cyclopentanone | 43.7 |
| 30. | 35.07 | 35.07 | Eicosane | 22.3 |
| 31. | 37.06 | 37.06 | Heneicosane | 18.4 |
| 32. | 38.05 | 38.05 | Heneicosane, 5-methyl- | 19.3 |

**Table S2.** Probable major compounds presents in plant extract and biosynthesized nanoparticles obtained by GC-MS/MS analysis.

**3. Supplementary Notes**

**Effect of parameters on synthesis of nanoparticle catalysts.** UV–vis spectroscopy is a high sensitive characterization technique to investigate the effect of parameters in the formation of nanoparticle (NP) catalysts with maximum absorbance peaks (λ_max_). Due to the surface plasmon resonance (SPR) of NPs absorption peak intensity varies and the overall synthesis conditions could be optimized.

*Effect of extract.* Effect of different concentration of plant extract on biosynthesis of NPs was studied. The synthesis of AgNPs was increased with the concentration of plant extract from 0.5% to 2%, while the reaction could not proceed at higher concentration (3%) as indicated by appearance of blackish colour of the solution (Fig. S1a). AuNPs exhibited almost similar trend to that of AgNPs and exhibited dark blue color of the solution at 3% (Fig. S1b). The color intensity of Au–Ag NPs solution also increased with concentration of plant extract up to 2% (Fig. S1c). Among the different concentration of extract, the SPR band intensity was maximum at 2% for all the NPs (Fig. 3a). Therefore, 2% leaf extract concentration appeared quite precise for maximum production of NPs for synthesis of NPs. The earlier reports suggested 5% leaf extract concentration for 100% conversion to AgNPs and AuNPs^1,2^.

*Effect of salt(s).* Different concentration of precursor salt was tried to explore the effect of precursor salt concentration on rate of formation of NPs. In case of AgNPs, color intensity increased steadily from 0.5 to 5 mM AgNO_3_ (Fig. S2a), but no significant increase in the SPR intensity was observed above 5 mM salt. Therefore, 5 mM AgNO_3_ appeared to be the best concentration for maximum formation of AgNPs (Fig. 3b). Above 5 mM salt concentration, the availability of the plant biomolecules may be insufficient to prevent the particles from aggregation as reported earlier for biosynthesis of AgNPs using *Pongamia pinnata* leaf extract^3^. For synthesis of AuNPs, the optimum concentration of chloroauric acid (HAuCl_4_.H_2_O) was 3 mM as indicated by formation of dark-purple color of the solution the intensity of which gradually increased from 1.0 mM (Fig. S2b). Thereafter, the intensity of SPR decreased up to 5 mM (turned to peach color) which indicates reaction saturation at 3 mM. Previous study also indicated the similar trend in biosynthesis of AuNPs using *Aspergillum* sp. WL–Au cell–free extracts^4^ with 3 mM chloroauric acid. The total concentrations of chloroauric acid and silver nitrate (1:1) used for synthesis of bimetallic Au–Ag NPs varied from 0.5-5.0 mM. Au–Ag NPs could not be synthesized using 2-5 mM of precursor salt(s), where the solution turned deep blue color to peach color due to precipitation of AgCl (Fig. S2c). SPR peak intensity of Au–Ag NPs using 1.0 mM precursor salt(s) was higher than that in 0.5 mM (Fig. 3b). Therefore, the optimum concentration of chloroauric acid and silver nitrate appeared to be 1 mM (0.5 mM each) for synthesis of Au–Ag NPs.

*Effect of Temperature.* Temperature is one of the key parameters that affect the synthesis of NPs. SPR peak intensity of AgNPs was increased with temperature due to slow reduction of Ag^+^, and the maximum formation of AgNPs was observed at 80 °C (Figs 3c and S3a)_._ The rate of reaction for synthesis of Au–Ag NPs was slow at 5 and 15 °C and further heating increased the rate of formation of NPs (Fig. S3c). The maximum SPR peak intensity of Au–Ag NPs was at 70 °C indicating reaction saturation (Fig. 3c). The formation of NPs at elevated temperature of the reaction mixture has also been reported earlier^5,6^. However, AuNPs didn’t show a detectable SPR peak at 5, 15 and 25 °C due to slow reaction rate. At this temperature, the solutions turned to light yellow color (Fig. S3b). The highest SPR peak intensity for AuNPs appeared at 50 °C (Fig. 3c) and above this temperature the reaction mixture tends to precipitate (Fig. S3b) indicate increase in particle size^7^.

*Effect of time.* Reaction time is very crucial for NPs synthesis. The color intensity of the reaction mixtures increased steadily over time and was distinct after 10 min for both AgNPs and Au–Ag NPs (Figs S4a and S4c) and after 30 min for AuNPs (Fig. S4b) signifying more homogeneity of the NPs. The synthesis of AgNPs was increased up to 45 minute and thereafter there was no measurable increase in SPR peak intensity (Fig. 3d). The formation of AuNPs was indicated by change in color from pale yellow to dark purple (Fig. S4b) with the highest peak intensity appeared at 75 min (Fig. 3d). The SPR peak intensity of Au–Ag NPs was also increased with time and was maximum at 60 min (Fig. 3d) indicating stability of Au–Ag NPs. Therefore, the present method is faster (< 1.5 h) than those reported for synthesis of Au, Ag and Au–Ag NPs (1.5-5.0 h or more) using plant extracts^8,9,10,11^.

*Effect of pH.* A wide pH (5.0 to 13.0) range was tested to understand the effect of pH on NPs formation. No color was developed for AgNPs formation at acidic pH–5.0 (Fig. S5a). The reaction started at pH–6.0 and AgNPs exhibited highest SPR peak intensity at pH–12.0 (Fig. 3e)^12^. The color developed for Au–Ag NPs synthesis is presented in Fig. S5c and the maximum SPR peak intensity appeared at pH–12.0 (Fig. 1f). The plant bio-molecules responsible for formation of NPs, might have been activated under strong alkaline conditions^13^ resulting in more efficient reduction of Ag^+^. In case of AuNPs synthesis, the intensity of purple color increased to dark–purple with increase in pH from 5.0 to 9.0 (Fig. S5b) and AuNPs were not synthesized above pH–9.0. The SPR peak intensity indicated pH–8.0 as optimum for synthesis of AuNPs (Fig. 3e). Thus the reaction rate for AuNPs was faster under weak alkaline medium rather than strong alkaline medium^4,13^. The difference in optimum pH condition for synthesis of AuNPs from that of Ag and Au–Ag NPs might be due to the involvement of different plant bio-molecules for their synthesis^14^.

**4. Supplementary References**

1. Song, J. Y. & Kim, B. S. Rapid biological synthesis of silver nanoparticles using plant leaf extracts. *Bioprocess Biosyst. Eng.* **32**, 79–84, <https://doi.org/10.1016/j.colsurfb.2012.09.007> (2009).

2. Song, J. Y., Jang, H–K. & Kim, B. S. Biological synthesis of gold nanoparticles using *Magnolia kobus* and *Diopyros kaki* leaf extracts. *Process Biochem.* **44**, 1133–1138, https://doi.org/10.1007/s00449-008-0224-6 (2009).

3. Priya, R. S., Geetha, D. & Ramesh, P. S. Antioxidant activity of chemically synthesized AgNPs and biosynthesized Pongamia pinnata leaf extract mediated AgNPs – A comparative study. *Ecotoxicol. Environ. Saf*. **134**, 308–318, <https://doi.org/10.1016/j.ecoenv.2015.07.037> (2016).

4. Qu, Y. et al. Biosynthesis of gold nanoparticles by Aspergillum sp. WL–Au for degradation of aromatic pollutants. *Physica. E Low dimens. System. Nanostruct*. **88**, 133–141, <https://doi.org/10.1016/j.physe.2017.01.010> (2017).

5. Sarkar, S., Jana, A. D., Samanta, S. K. & Mostafa, G. Facile synthesis of silver nano particles with highly efficient anti–microbial property. *Polyhedron,* **26**, 4419–4426, <https://doi.org/10.1016/j.poly.2007.05.056> (2007).

6. Kumar, D., Kumar, G., Das, R. & Agrawal, V. Strong larvicidal potential of silver nanoparticles (AgNPs) synthesized using *Holarrhena antidysenterica* (L.) Wall. bark extract against malarial vector. *Anopheles stephensi* Liston. *Process Saf. Environ, Prot..* **116**, 137–148, <https://doi.org/10.1016/j.psep.2018.02.001> (2018).

7. Wadhwani, S. A., Shedbalkar, U. U., Singh, R., Karve, M. S. & Chopade, B. A. Novel polyhedral gold nanoparticles: green synthesis, optimization and characterization by environmental isolate of *Acinetobacter* sp. SW30. *World J. Microbiol. Biotechnol*. **30**, 2723–2731, <https://doi.org/10.1007/s11274-014-1696-y> (2014).

8. Jacob, J., Mukherjee, T. & Kapoor, S. A simple approach for facile synthesis of Ag, anisotropic Au and bimetallic (Ag/Au) nanoparticles using cruciferous vegetable extracts. *Mater. Sci. Eng. C*. **32**, 1827–1834, <https://doi.org/10.1016/j.msec.2012.04.072> (2012).

9. Salunke, G. R. *et al*. Rapid efficient synthesis and characterization of silver, gold, and bimetallic nanoparticles from the medicinal plant *Plumbago zeylanica* and their application in biofilm control. *Int. J. Nanomed*. **9**, 2635–2653, <https://doi.org/10.2147/IJN.S59834> (2014).

10. Tamulya, C., Hazarika, M., Borah, S. C., Das, M. R. &. Boruah, M. P., In situ biosynthesis of Ag, Au and bimetallic nanoparticles using *Piper pedicellatum* C.DC: Green chemistry approach. *Colloids Surf. B: Biointerfaces* **102**, 627– 634, <https://doi.org/10.1016/j.colsurfb.2012.09.007> (2013).

11. Sastry, M., Shankar, S. S., Akhilesh, R. & Absar, A. Rapid synthesis of Au, Ag, andbimetallic Au core–Ag shell nanoparticles using Neem (Azadirachta indica) leaf broth. *J. Colloid Interface Sci..* **275**, 496–502, <https://doi.org/10.1016/j.jcis.2004.03.003> (2004).

12. Gou, Y., Zhou, R., Ye, X., Gao, S. & Li, X., Highly efficient in vitro biosynthesis of silver nanoparticles using *Lysinibacillus sphaericus* MR–1 and their characterization. *Sci. Technol. Adv. Mater*. **16**, 015004, <https://doi.org/10.1088/1468-6996/16/1/015004> (2015).

13. Parial, D. & Pal, R., Biosynthesis of monodisperse gold nanoparticles by green alga *Rhizoclonium* and associated biochemical changes. *J. Appl. Phycol*. **27**, 975-984, https://doi.org/10.1007/s10811-014-0355-x (2015).

14. Hamid, A. A. A., Al–Ghobashy, M., Fawzy, M., Mohamed, M. & Abdel–Mottaleb, M. M. S. A. Phytosynthesis of Au, Ag and Au–Ag bimetallic nanoparticles using aqueous extract of sago pondweed (*Potamogeton pectinatus* L.). *ACS Sustain. Chem. Eng.***1**, 1520–1529, <https://doi.org/10.1021/sc4000972> (2013).
